# Increased weight loading reduces body weight and body fat in obese subjects – A proof of concept randomized clinical trial

**DOI:** 10.1016/j.eclinm.2020.100338

**Published:** 2020-04-30

**Authors:** Claes Ohlsson, Edwin Gidestrand, Jacob Bellman, Christel Larsson, Vilborg Palsdottir, Daniel Hägg, Per-Anders Jansson, John-Olov Jansson

**Affiliations:** a Centre for Bone and Arthritis Research, Department of Internal Medicine and Clinical Nutrition, Institute of Medicine, the Sahlgrenska Academy at University of Gothenburg, Gothenburg, Sweden; b Region Västra Götaland, Sahlgrenska University Hospital, Department of Drug Treatment, Gothenburg, Sweden; c Department of Physiology, Institute of Neuroscience and Physiology, The Sahlgrenska Academy at the University of Gothenburg, Gothenburg, Sweden; d Department of Food and Nutrition, and Sport Science, University of Gothenburg, Sweden; e Wallenberg Laboratory, Department of Molecular and Clinical Medicine, Institute of Medicine, the Sahlgrenska Academy, University of Gothenburg, Gothenburg, Sweden; f Gothia Forum, Region Västra Götaland, Sahlgrenska University Hospital, Gothenburg, Sweden

## Abstract

**Background:**

Recently we provided evidence for a leptin-independent homeostatic regulation, *the gravitostat*, of body weight in rodents. The aim of the present translational proof of concept study was to test the gravitostat hypothesis in humans.

**Methods:**

We conducted a randomized controlled single center trial (ClinicalTrial.gov number, NCT03672903), to evaluate the efficacy of artificially increased weight loading on body weight in subjects with mild obesity (BMI 30–35 kg/m^2^). Subjects were either treated with a heavy (=high load; 11% of body weight) or light (=low load; 1% of body weight) weight vest for eight hours per day for three weeks. The primary outcome was change in body weight. Secondary outcomes included change in body fat mass and fat-free mass as measured using bioelectrical impedance analysis.

**Findings:**

In total 72 participants underwent randomization and 69 (36 high load and 33 low load) completed the study for the primary outcome. High load treatment resulted in a more pronounced relative body weight loss compared to low load treatment (mean difference -1.37%, 95% confidence interval (CI), -1.96 to -0.79; *p* = 1.5 × 10^−5^). High load treatment reduced fat mass (-4.04%, 95% CI, -6,53 to -1.55; *p* = 1.9 × 10^−3^) but not fat free mass (0.43%, 95% CI, -1.47 to 2.34; *p* = 0.65) compared to low load treatment.

**Interpretation:**

Increased weight loading reduces body weight and fat mass in obese subjects in a similar way as previously shown in obese rodents. These findings demonstrate that there is weight loading dependent homeostatic regulation of body weight, the gravitostat, also in humans.

**Funding:**

Funded by Jane and Dan Olsson (JADO) Foundation, the Torsten Söderberg Foundation, The Knut and Alice Wallenberg's Foundation and the Novo Nordisk Foundation.

**Research in context**

***Evidence before this study***

**Until recently, the only known homeostatic regulator of fat mass was the fat-derived hormone leptin. However, we have recently published evidence that there is a loading dependent homeostatic regulation of body weight and fat mass, named the gravitostat, supported by the finding that increased loading using weight capsules reversibly decreased body weight and fat mass in rodents. The aim of the present proof of concept translational study was to investigate if artificially increased weight loading decreases biological body weight also in obese humans. We searched PubMed for studies published before Jan 7th, 2020 with the search criteria "weight loading" AND "randomized clinical trial". The search found no previous study.**

***Added value of this study***

**The present randomized clinical trial demonstrates that increased weight loading reduces body weight and fat mass in obese subjects.**

***Implications of all the available evidence***

**Increased weight loading reduces body weight and fat mass in obese subjects in a similar way as previously shown in obese rodents. These findings demonstrate that there is a weight loading dependent homeostatic regulation of body weight, the gravitostat, also in humans.**

Alt-text: Unlabelled box

## 1 Introduction

Obesity is a growing problem worldwide, and it is associated with increased mortality and morbidity [1]. At present, there are few effective pharmacological treatments available for obesity [2,3]. One possible reason for this is insufficient basic information about the regulation of body weight and fat mass. Many diseases can be understood as disturbances of homeostatic mechanisms. The concept of homeostasis and its name were introduced in the 1800s and early 1900s by Claude Bernard and Walter B. Cannon [4], [5], [6], and has turned out to be advantageous for the understanding of both physiology and disease mechanisms. More than a quarter of a century ago Friedman and colleagues discovered the fat-derived hormone leptin [7], and, until recently, thisdd was the only known homeostatic regulator of fat mass. The importance of leptin is clear from the finding that genetically caused lack of leptin results in severe obesity that can be reversed by leptin treatment in both experimental animals [8,9] and humans [10]. In line with this, it has been demonstrated that blockade of endogenous leptin increases body fat mass to the same extent in mice with diet-induced obesity as in lean mice [11]. However, in most cases of obesity, the endogenous serum leptin levels are high, and there is limited effect by leptin treatment when evaluated in animal studies or randomized clinical trials, indicating that other homeostatic mechanisms also might contribute [12], [13], [14], [15], [16], [17]. We have recently published evidence that there is a loading dependent homeostatic regulation of body weight and fat mass, named the gravitostat, supported by the finding that increased loading using weight capsules reversibly decreased body weight and fat mass in rodents [18], [19], [20], [21]. Importantly, studies using leptin-deficient obese (Ob/Ob) mice demonstrated that increased loading regulated fat mass independently of fat-derived leptin, revealing two independent negative feedback systems for fat mass regulation in rodents [18,19]. In addition, we observed that the effects of increased loading on body weight and fat mass were most pronounced in obese rodents and we proposed that the long-sought anti-obesity signal acting mainly at a comparatively high body weight may involve the gravitostat [15,17,19]. The aim of the present proof of concept translational study was to investigate if artificially increased weight loading decreases biological body weight also in obese humans.

## 2 Methods

### 2.1 Trial design and oversight

This study (Effect of different weight Vests on body weight in Obese subjects; *EVO*; ClinicalTrial.gov number, NCT03672903) in subjects with mild obesity (BMI > 30 and ≤ 35 kg/m^2^) was a randomized single center trial, to evaluate the efficacy on percentage change from baseline in body weight of a treatment with a heavy weight vest (=high load) compared with treatment with a light weight vest (=low load). We screened 99 subjects at a single center (Gothia Forum at Sahlgrenska University hospital in Gothenburg, Sweden). A total of 69 eligible participants were randomly assigned in a 1:1 ratio to receive the low load or high load treatment. The 1:1 randomization was performed by one of the investigators using a dice. When we got values 1–3 the participant was randomized to heavy weight vest, and when we got values 4–6 the participant was randomized to a light weight vest. Blinding was not possible as both the investigators and the participants could feel how heavy the selected weight vest was. The study was externally monitored by two monitors appointed by the investigator. The monitors had access to all the data and performed monitoring visits on site before, during and after data collection.

The trial protocol was reviewed and approved by the Ethics committee in Gothenburg (Dnr 652–18). All patients provided written informed consent before participation. The design of the study was performed by EG, PAJ, CO and JOJ. EG, PAJ, CO and JOJ take primary responsibility for the data and analyses and for the fidelity of the study to the protocol. The first and last author wrote the first draft of the manuscript. All the authors contributed to subsequent drafts of the manuscript and made the final decision to submit the manuscript for publication.

### 2.2 Trial participants

Healthy men and women with mild obesity (degree 1 obesity, BMI > 30 and ≤ 35 kg/m^2^), between 18 and 70 years of age, willing to comply with the study protocol, with normal or clinically non-significant aberrations of screening blood- and urine samples and signed informed consent were eligible for participation. The exclusion criteria were the following: chronic disease that hardens the participation in the study as judged by the investigator, chronic pain, regular consumption of medicine or natural supplements that affect weight, inhibit physical activity or increase the risk of adverse effects as judged by the investigator, bariatric-metabolic operation, reduced mobility, pregnancy, change in body weight of 5 kg or greater during the past 3 months, a greater than 1.5 kg difference in body weight between the screening visit and the baseline visit, drastic change in lifestyle during the last three months (change in physical activity or nicotine or alcohol use), or apparent risk of not being able to comply with the study protocol for any reason as judged by the investigator.

### 2.3 Procedures

During a screening visit, signed informed consents for participation in the study were collected and the subjects were checked for compliance with the eligibility criteria. Eligible subjects were scheduled for a baseline visit (baseline visit; randomization) one week after the screening visit. It was then checked that they had not substantially altered their body weight (≤1.5 kg) compared with the screening visit. It was also then further established that they were able to adhere to the study protocol. Eligible subjects were then at the baseline visit randomly assigned, in a 1:1 ratio, to receive heavy or light body weight loading. The heavy loading consisted of a weight vest with a weight corresponding to 11% of the subject's body weight (PRF Weight vest, Casall, Norrköping, Sweden) and the light loading consisted of a weight vest from the same manufacturer (PRF Weight vest, Casall, Norrköping, Sweden) with identical appearance with a weight corresponding to 1% of the subject's body weight. 11% and 1% of body weight were chosen as we aimed to have 10% difference between the treatment groups but still not have too heavy weight vests to minimize possible side effects. The participants were asked to use the weight vest for at least eight hours per day for three weeks. The participant recorded daily the time using the weight vest and the time using the weight vest standing. Compliance to wearing the weight vest was evaluated using the participants written recordings of the time using the weight vest. Subjects were encouraged to continue with their normal lifestyle except for the extra amount of standing time each day. An additional visit was conducted at the end of the study three weeks after the baseline visit. Furthermore, the study participants were contacted by phone one and two weeks after the baseline visit to confirm that they were using the weight vest according to the protocol and to collect information on any adverse events. Body weight was measured at the screening visit, at the baseline visit and at the end of the study three weeks after the baseline visit using the same high quality scale (MC-180MA, Tanita; coefficient of variation (CV) < 0.2%) for all visits and all subjects. The same equipment was also used for bioelectrical impedance analysis (BIA) of total body fat mass, fat free mass and fat percentage at the same visits as the body weight were measured (Fat mass, CV 1.48%; Fat free mass CV 0.60%). Body composition was measured by BIA and not using a gold standard method such as dual energy X-ray absorptiometry (DXA). However, the DXA and BIA methods display an excellent correlation and for measurement of changes in body composition within a certain individual, the BIA is reported to be a method with good reliability [22], [23], [24]. Serum was collected and immediately frozen at baseline and at the visit after three weeks of loading treatment and the samples were kept frozen at −80°C until analysis. Levels of insulin (Mercodia; product number 10–1113–01, intra-assay variability 3.3%; Uppsala, Sweden), leptin (R&D Systems; product number DLP00, intra-assay variability 3.2%; Minneapolis, MN, USA) and adiponectin (As One International; product number K1001-1, intra-assay variability 3.6%; Santa Clara, CA, USA) were measured by commercial ELISA kits in fasting serum samples. Levels of lipids (Total cholesterol, intra-assay variability 1.3%; Low Density Lipoprotein [LDL], intra-assay variability 2.0%; High Density Lipoprotein [HDL], intra-assay variability 1.6%; and Triglycerides [TG], intra-assay variability 1.5%) were analysed at the central laboratory of Sahlgrenska University Hospital (Gothenburg, Sweden) using the Cobas analysis platform (Roche, Basel, Switzerland). Plasma glucose levels were analysed directly using a glucometer (HemoCue Glucose 201 RT; Ängelholm, Sweden; intra-assay variability 2.3%).

**Fig. 1:**
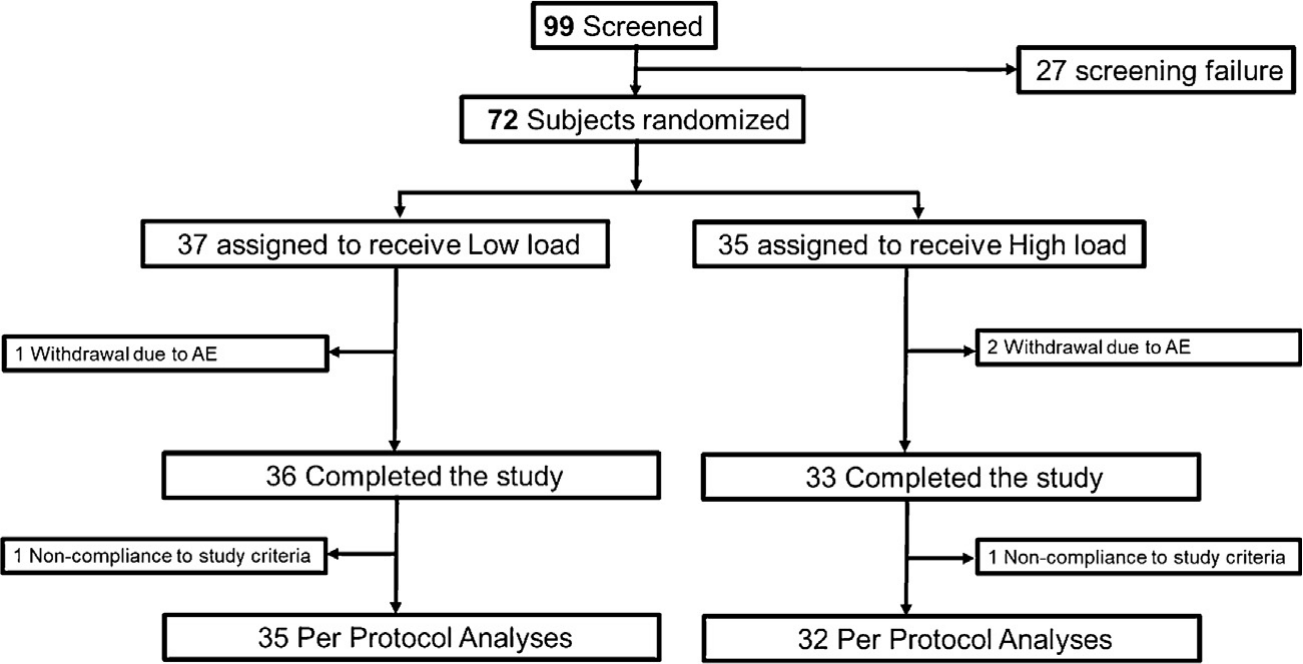
**CONSORT Diagram describing enrolment and randomization.** The 27 subjects with screening failure did not meet all inclusion criteria and/or did meet at least one of the exclusion criteria as described in Methods.

To estimate daily energy intake, a validated food questionnaire called “Short Dietary Questionnaire” (SDQ) [25] was filled out by patients every week including the week between the screening and the baseline visit, during week one of the study, during week two of the study and during week three of the study. Adverse events spontaneously reported by the subjects, observed or elicited based on non-leading questions by the investigator were collected from the time of signing the informed consent until completion of the study. All study participants were instructed by a physician to refrain from excessive amounts of alcohol (maximum 1 liter, 11% alcohol or equivalent for a full week) or using any drugs besides smoking or snuff. To be included in the per protocol analyses, the study subjects should not deviate more than 20% (1.6 h) from the requirement of using the weight vest at least eight hours per day (Fig. 1).

### 2.4 Outcomes

The primary endpoint was the percent change from baseline in body weight in the high load group compared with low load group. The secondary endpoints included the percent change from baseline in fat mass, fat-free mass, energy intake, serum leptin, serum insulin, serum LDL, serum HDL, serum TG and plasma glucose in the high load group compared with low load group. The key exploratory endpoints included the percent change from baseline in total body fat percentage, serum adiponectin, serum total cholesterol and HOMA-index in the high load group compared with low load group. For the body weight and the BIA analysed parameters reflecting body composition, the absolute changes from baseline for the high load group compared with low load group were also evaluated as exploratory endpoints.

### 2.5 Statistical analysis

Power calculations demonstrated that a total of 100 subjects would be required to be screened to identify a statistically significant (*p*<0.05) effect of 1.6% difference in relative body weight change between three weeks and baseline, when comparing the high load with the low load group with 80% power. These calculations allowed a screening failure of approximately 20% and were based on a standard deviation of 2.55% for change in body weight comparing three weeks with baseline.

The statistical analyses were performed according to a statistical analysis plan developed before the end of the study. The difference between the treatment groups for all parameters were tested by analysis of covariance (ANCOVA) with relative change from baseline to three weeks as dependent variable, treatment group as fixed effect and age, sex, baseline BMI, vest exposure (h/day) and standing% when using the weight vest (=vest time standing/total vest time *100) as covariates. From these ANCOVA models adjusted for covariates, least square means (LSM) with 95% confidence intervals (CI) are presented. The p-values given for the within group comparison (three weeks vs baseline) of the different parameters were calculated using Wilcoxon signed rank-sum test. The statistical analyses of body weight were conducted for all randomized subjects with body weight measurements available both at baseline and at three weeks (69 participants, 36 low load and 33 high load; Fig. 1). Besides for the three subjects who discontinued the study due to adverse events, there were no missing data for body weight or the parameters reflecting body composition using BIA. There were few data points missing for serum/plasma markers and food intake and no imputation of missing data for these parameters was performed. The number of subjects available for statistical analyses for each of these parameters are given in Table S4.

For comparisons of adverse events between the treatment groups (Table 3), Fisher´s exact test was used. Analyses of adverse events and safety included all participants who underwent randomization and had used the weight vest for at least one day (Fig. 1; Table 3).

### 2.6 Role of funding

This research was funded by grants from Jane and Dan Olsson (JADO) Foundation, the Torsten Söderberg Foundation, The Knut and Alice Wallenberg's Foundation and the Novo Nordisk Foundation. The funders had no role in design and conduct of the study; collection, management, analysis and interpretation of the data; or in preparation, review or approval of the manuscript.

## 3 Results

In total, 99 subjects were screened and 72 subjects who fulfilled the screening criteria and agreed to participate in the study were randomized to either low load or high load (Fig. 1). Study subjects were included between September 2018 and January 2019 and completed the study between October 2018 and February 2019. A total of 36 of the 37 participants who were assigned to the low load vest (97%) and 33 of the 35 participants who were assigned to the high load vest (94%) completed the two body weight recordings required for the primary outcome relative change in body weight (baseline and week 3; Fig. 1). Two participants, one in the low load group (6.22 h vest time/day) and one in the high load group (5.99 h vest time/day), deviated more than 20% from the required 8 h use of the weight vest per day and these were consequently excluded in the per protocol analyses (=PPA, *n* = 67; 35 low load and 32 high load; Fig. 1). The study groups were well balanced with regards to baseline characteristics except that subjects in the high load group had lower serum leptin levels than subjects in the low load group (*p* = 0.028; Table 1; Supplemental Table 1). Neither the average hours of vest exposure nor the percent standing time during the vest exposure differed between the two treatment groups (Table 1).

**Table 1:** Baseline characteristics.

Characteristic	Low Load	High load	p-value
	(*N* = 37)	(*N* = 35)	
Age (years)	48.5 ± 13.1	50.4 ± 10.6	NS
Females (%)	30 (81%)	24 (69%)	NS
Height (cm)	169 ± 8	172 ± 11	NS
Weight (kg)	93.2 ± 10.0	95.5 ± 13.4	NS
BMI (kg/m^2^)	32.3 ± 1.7	32.2 ± 1.4	NS
Vest exposure (h)	9.0 ± 1.4	8.4 ± 1.4	NS
Standing (%)	57.0 ± 19.4	64.9 ± 18.4	NS
Fat percent (%)	38.3 ± 5.2	36.6 ± 6.0	NS
Fat mass (kg)	35.3 ± 4.6	34.5 ± 5.0	NS
Fat free mass (kg)	57.6 ± 9.9	60.9 ± 12.8	NS
Serum markers	(*N* = 34)	(*N* = 33)	
Total Cholesterol (mmol/L)	5.1 ± 0.9	5.5 ± 1.2	NS
HDL Cholesterol (mmol/L)	1.4 ± 0.3	1.4 ± 0.4	NS
LDL Cholesterol (mmol/L)	3.5 ± 0.8	3.8 ± 1.1	NS
Triglycerides (mmol/L)	1.3 ± 0.5	1.5 ± 0.8	NS
Leptin (ng/ml)	44.0 ± 21.2	33.0 ± 18.7	0.028
Adiponectin (ng/ml)	2.4 ± 1.0	2.6 ± 1.3	NS
Insulin (mU/l)	12.4 ± 7.3	11.4 ± 7.8	NS
HOMA index	2.9 ± 1.7	2.7 ± 2.1	NS
	(*N* = 36)	(*N* = 35)	
Plasma Glucose (mmol/L)	5.3 ± 0.5	5.2 ± 0.8	NS
	(*N* = 34)	(*N* = 32)	
Food intake (Kcal/day)	1806 ± 939	1771 ± 709	NS

Values are given as mean ± SD or *n* (%) for all randomized subjects. For comparisons between groups, Fisher´s exact test was used for dichotomous variables, *t*-test was used for normally distributed continuous parameters and Mann-Whitney *U* test was used for non-normally distributed parameters. BMI = body mass index, NS = non-significant.

### 3.1 Primary outcome

The primary analysis was performed for all randomized subjects who completed the two body weight recordings (baseline and week three) required for the primary outcome relative change in body weight (*n* = 69). The body weight loss (relative change after 3 weeks) was significant in the high load group (−1.68%, 95% confidence intervals (CI), −2.09 to –1.27) but not in the low load group (−0.31%, 95% CI, −0.70 to 0.08; Table 2). High load treatment resulted in a more pronounced relative body weight loss compared to low load treatment (*=primary outcome, p* = 1.5 × 10^−5^, mean difference −1.37%, 95% CI, −1.96 to −0.79; Table 2). Very similar results were observed for relative change in body weight in the per protocol analyses (*n* = 67; mean difference −1.34%, 95 CI, −1.94 to −0.74; Supplemental Table 2).

**Table 2:** Analyses of the relative and absolute changes in the primary and secondary outcomes for all subjects who completed the study.

	Within group comparison			
	Low Load (*N* = 36)	High load (*N* = 33)	Difference between groups	P ANCOVA
***Primary outcome***				
*Relative change*				
Body weight (%)	−0.31 (−0.70 to 0.08)	−1.68 (−2.09 to −1.27)***	−1.37 (−1.96 to −0.79)	1.5E-05
***Secondary outcomes***				
*Relative change*				
Fat mass (%)	−0.79 (−2.46 to 0.88)	−4.82 (−6.57 to −3.08)***	−4.04 (−6.53 to −1.55)	1.9E-03
Fat free mass (%)	−0.04 (−1.32 to 1.24)	0.39 (−0.95 to 1.73)	0.43 (−1.47 to 2.34)	0.65
Fat percent (%)	−0.48 (−2.17 to 1.20)	−3.18 (−4.95 to −1.42)***	−2.70 (−5.21 to −0.19)	0.035
*Absolute change*				
Body weight (kg)	−0.30 (−0.66 to 0.05)	−1.61 (−1.98 to −1.24)***	−1.31 (−1.84 to −0.78)	6.3E-06
Fat mass (kg)	−0.22 (−0.82 to 0.37)	−1.73 (−2.36 to −1.10)***	−1.51 (−2.40 to −0.61)	1.3E-03
Fat free mass (kg)	−0.08 (−0.74 to 0.58)	0.12 (−0.57 to 0.81)	0.20 (−0.79 to 1.18)	0.69
Fat percent (%)	−0.16 (−0.83 to 0.51)	−1.25 (−1.95 to −0.54)***	−1.09 (−2.09 to −0.08)	0.034

The primary outcome was the relative change after 3 weeks in body weight. Results are presented as least square means with 95% confidence intervals for all randomized subjects with body weight available both at baseline and at 3 weeks treated with light weight vest (Low load; *n* = 36) or heavy weight vest (High load, *n* = 33). The between group p-values (High load vs Low load) given within the table are calculated using analysis of covariance (ANCOVA) adjusted for age, sex, baseline BMI, Vest exposure (h) and standing % with vest. *** = *p*<0.001 for within group comparison (Three weeks vs baseline) using Wilcoxon signed rank-sum test.

**Fig. 2:**
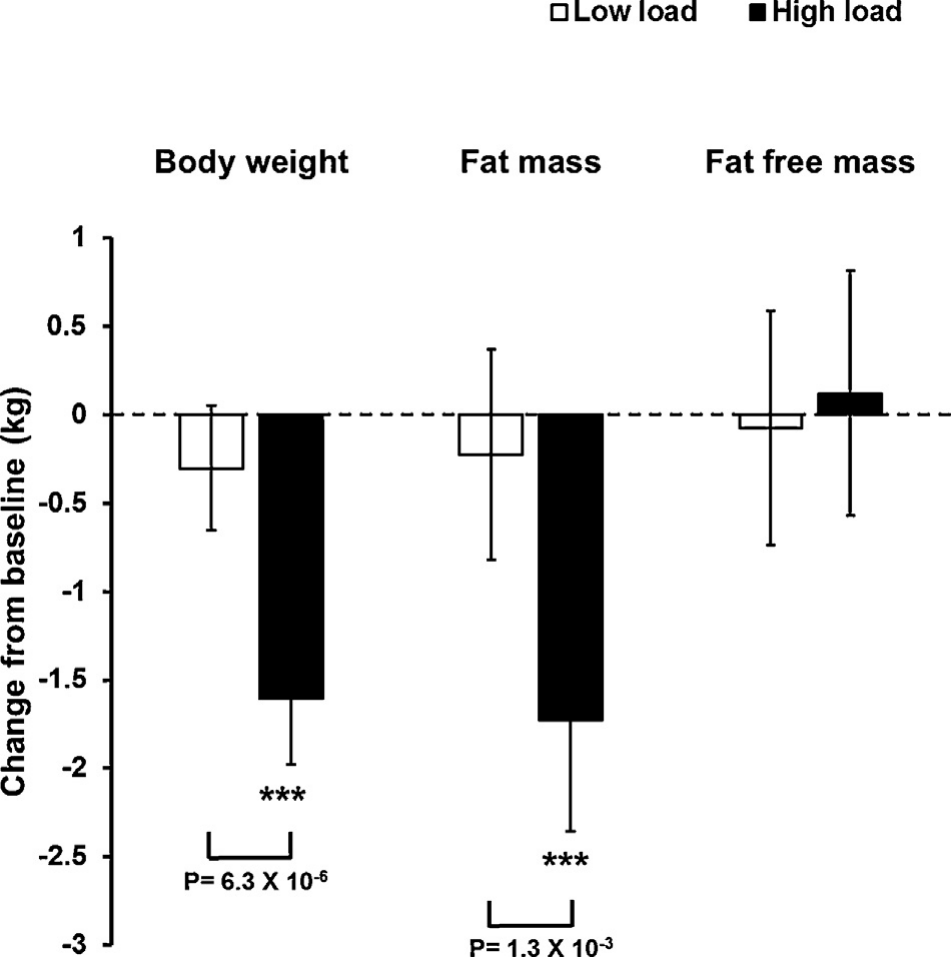
**Change in body weight, fat mass and fat free mass at 3 weeks vs baseline for all subjects who completed the study.** Results are presented as least square means with 95% confidence intervals for all randomized subjects with body weight available both at baseline and at 3 weeks treated with light weight vest (Low load; *n* = 36) or heavy weight vest (High load, *n* = 33). The between group p-values (High load vs Low load) given within the figure are calculated using analysis of covariance (ANCOVA) adjusted for age, sex, baseline BMI, Vest exposure (h) and standing % with vest. *** = *p*<0.001 for within group comparison (Three weeks vs baseline) using Wilcoxon signed rank-sum test.

Analysis of the absolute change in body weight showed that the high load treatment reduced the body weight with in average 1.31 kg (95% CI, −1.84 to −0.78) compared to the low load treatment (Table 2, Fig. 2) and sex stratified analyses revealed that the high load treatment reduced the absolute body weight both in women (−1.36 kg, 95% CI, −2.00 to −0.71) and men (−1.17 kg, 95% CI, −1.17 to −0.14) compared with the low load treatment (Supplemental Table 3).

### 3.2 Secondary/exploratory outcomes

Separate analyses of the secondary outcomes fat mass and fat free mass at baseline and at week three for all randomized subjects who completed the study revealed that the observed treatment effect of high load vs low load on change in body weight was caused by a reduction of fat mass (between group difference in relative change in fat mass −4.04%, 95% CI, −6,53 to −1.55) while fat free mass was unaffected (between group difference in relative change in fat free mass 0.43%, 95% CI, −1.47 to 2.34). Analyses of the absolute change in fat mass demonstrated that high load treatment reduced fat mass by 1.73 kg (95% CI, 2.36 to 1.10 kg) while no statistically significant effect was observed by low load treatment (Fig. 2, Table 2). The within group difference for relative change in fat percent between week 3 and baseline was significant in the high load group (−3.18%, 95% CI, −4.95 to – 1.42) but not in the low load group (−0.48%, 95% CI, −2.17 to 1.20), resulting in a significant between group difference (−2.70%, 95% CI, −5.21 to −0.19; Table 2). Very similar between group results for changes in fat mass and fat percent were observed in the per protocol analyses (Supplemental Table 2).

Exploratory mechanistic outcome analyses did not observe any treatment related between group differences for serum/plasma parameters (total cholesterol, HDL cholesterol, triglycerides, leptin, adiponectin, glucose, insulin or calculated HOMA index) except for a modest reduction in relative change of LDL cholesterol in the high load group compared with the low load group (relative change −7.84%, 95% CI −14.15 to −1.54, supplemental Table 4). Within group analyses revealed that the high load treatment reduced serum leptin levels significantly in the high load treatment group (−13.59%, 95% CI −22.33 to −4.84) but not in the low load group (−6.37%, 95% CI, −14.98 to 2.24; supplemental Table 4). No statistically significant treatment effect was observed on self-reported food intake (Energy intake/day; Supplemental Table 4).

### 3.3 Adverse effects and safety

No serious adverse event was reported in any of the treatment groups. One participant in the low load group (myalgia) and two participants in the high load group (pain in lower extremity and influenza) discontinued the trial due to adverse events (Table 3). The proportion of participants reporting any adverse event was higher in the high load group (37.1%) compared with the low load group (16.2%) and this difference was the result of a higher proportion of musculoskeletal adverse events in the high load group (20.0%; 1 arthralgia, 2 myalgia, 2 pain in lower extremity and 2 Swelling of ankle and/or foot) compared with the low load group (2.7%; 1 myalgia). No significant between group difference was observed for any other adverse events.

**Table 3:** Self-reported adverse and serious adverse events among the study participants.

	Low load	High load	p-value
	(*N* = 37)	(*N* = 35)	
**Any adverse events, n (%)**			
*Musculoskeletal disorders*	1 (2.7)	7 (20.0)	*p* < 0.05
Arthralgia	0 (0)	1 (2.9)	NS
Myalgia	1 (2.7)	2 (5.7)	NS
Pain in lower extremity	0 (0)	2 (5.7)	NS
Swelling of ankle and/or foot	0 (0)	2 (5.7)	NS
*Other*	5 (13.5)	6 (17.1)	NS
Hyperhidrosis	3 (8.1)	0 (0)	NS
Decreased appetite	0 (0)	4 (11.4)	NS
Increased appetite	0 (0)	1 (2.9)	NS
Fatigue	1 (2.7)	1 (2.9)	NS
Uncomfortable feeling	1 (2.7)	0 (0)	NS
*Infections*	1 (2.7)	3 (8.6)	NS
Influenza	0 (0)	1 (2.9)	NS
Upper respiratory infection	1 (2.7)	2 (5.7)	NS
*Nervous system disorder*	0 (0)	1 (2.9)	NS
Migraine	0 (0)	1 (2.9)	NS
*Gastrointestinal disorder*	0 (0)	0 (0)	NS
*Heart and/or lung disorder*	0 (0)	0 (0)	NS
*Injury*	0 (0)	0 (0)	NS
**Adverse events leading to discontinuation of trial^#^, n (%)**			
*Musculoskeletal disorders*	1 (2.7)	1 (2.9)	NS
Myalgia	1 (2.7)	0 (0)	NS
Pain in lower extremity	0 (0)	1 (2.9)	NS
*Infection*	0 (0)	1 (2.9)	NS
Influenza	0 (0)	1 (2.9)	NS
#One participant in the low load group and two participants in the high load group discontinued the trial			
**Participants with any adverse event, n (%)**	6 (16.2)	13 (37.1)	*p* < 0.05
**Participants with any treatment-related adverse event, n (%)**	5 (13.5)	5 (14.3)	NS
**Number of treatment-related adverse events**	5	6	NS
**Any serious adverse event, n (%)**	0 (0)	0 (0)	NS
**Any treatment-related serious adverse event, n (%)**	0 (0)	0 (0)	NS

Adverse events are presented as number of events together with percentage for all subjects who were randomized.

## 4 Discussion

Previous studies demonstrate that increased loading reduces body weight and body fat mass in obese rodents [18,19,21]. We, herein, performed a proof of concept translational randomized clinical trial, evaluating the effect of increased loading in obese humans. The main finding was that high load treatment reduced body weight compared to low load treatment and that this was the result of a reduction in fat mass while fat-free mas was unaffected. Our interpretation of these results is that there is a weight loading dependent homeostatic regulation of body weight, the gravitostat, also in humans.

Although the between group effect of increased loading on the primary endpoint relative change in body weight was clearly statistically significant (*p* = 1.5 × 10^−5^), the effect size, 1.37% (=1.31 kg), was moderate. However, it should be emphasized that this was a proof of concept randomized clinical trial with a rather short (three weeks) duration. This is substantially shorter than that mostly used to evaluate a pharmaceutical treatment of obesity. A recent extensive meta-analysis of randomized controlled obesity trials revealed that 52 weeks’ treatment with the approved pharmacologic monotherapies of obesity liraglutide (−5.3 kg), orlistat (−2.6 kg), and lorcaserin (−3.2 kg) reduced the body weight 2–4 fold more than what the increased loading did in the present short-term three-week study [26]. Moreover, the effect of increased weight loading on body weight seems to be robust when comparing with the variable, often small, effects described in randomized controlled trials of exercise and other life style changes [27], [28], [29] as well as of leptin treatment on body weight in most cases of obesity [12], [13], [14], [15], [16].

We recently put forward the gravitostat hypothesis, that there is a loading dependent homeostat in the lower extremities regulating body weight [18,20]. This gravitostat would (together with leptin) ensure sufficient whole body energy depots but still protect land-living animals from becoming too heavy [[18], [19], [20],^30^]. We propose that the decrease in biological body weight is a compensatory effect to partly restore total body weight after increased weight loading. Thus, the present findings would reflect a loading dependent homeostatic regulation of body weight; the gravitostat.

Increased loading most likely to some extent increases energy expenditure to provide energy for an inevitable increase in physical workload. Especially when moving upward the energy expenditure must increase to gain potential energy, as it is proportional to the body mass and the acceleration caused by gravity. This mechanism involving increased energy expenditure could be regarded as a part of the gravitostat as it senses the body weight and adjusts the body mass [20]. In our previous experimental studies on loading in rodents we did not notice any significantly increased energy expenditure but instead a clearly reduced food intake [18]. Thus, the gravitostat may have the capacity to regulate both food intake and energy expenditure to maintain a constant body mass. In the present clinical trial, we did not observe any significant effect of increased loading on food intake in humans. As the variations are large and the reliability of self-reported data is questionable for food intake, we believe that the present negative findings on food intake should be further evaluated in larger studies using objectively determined food intake data. We tried to measure physical activity in the present study using accelerometers, but these analyses failed since the accelerometers broke down and very frequently reset themselves to 0 during the study. Thus, future detailed mechanistic studies should determine if the weight loading induced reduction in body weight and body fat in humans involves regulation of food intake, physical activity and/or energy expenditure.

Sex stratified analyses demonstrated that the effects of increased loading on both body weight and fat mass was significant in both genders and the effect sizes were of similar magnitudes for men and women, indicating that the loading dependent homeostatic regulation of body weight is not modulated by gender in humans.

No serious adverse event was reported in any of the treatment groups. However, an increased frequency of musculoskeletal adverse events was reported in the high load group compared with the low load group, most likely due to the intentionally increased axial load on the lower limbs in the high load group compared with the low load group. However, only one subject in each treatment group discontinued the study due to musculoskeletal adverse events. Further long-term studies should determine if the increased risk of musculoskeletal adverse effect is transient “*exercise induced soreness*” or long lasting. Besides musculoskeletal adverse events, there was no difference between the treatment groups for any other reported adverse event.

The strengths of the present proof of concept study are the randomized design with a pre-specified analysis plan and robust positive results for a clinically relevant primary outcome. We believe it is a strength that the control low load group was treated with an identical weight vest but with less weight added compared with the weight vest in the high load group. Nevertheless, as both the investigators and the participants could feel how heavy the selected weight vest was, blinding was unfortunately not possible. It is a limitation of the present study that the daily time of using the weight vest and the time using the weight vest standing were self-reported. The present short-term proof of concept study did not last for more than three weeks and, therefore, future long-term studies are warranted to determine if prolonged treatment results in a more pronounced weight loss or if the treatment effect is attenuated by time. In addition, it is a limitation with the present study that the secondary/exploratory analyses of serum markers and food intake did not reveal any clear underlying mechanism for the observed effect on body weight. However, the short duration of the study may make it difficult to observe effects of increased loading on secondary serum parameters that are affected by obesity. Furthermore, a modest but statistically significant loading induced reduction of serum LDL was actually observed. Although the present study revealed a robust loading induced reduction in overall fat mass as analysed by BIA, this technique cannot determine if this treatment mainly affected the metabolically active visceral fat, an independent risk factor for the metabolic syndrome and for cardiovascular disease [31], [32], [33], or mainly the subcutaneous fat. Future studies should evaluate the effect of increased weight loading on these two types of adipose tissue separately. Finally, the present study did only evaluate the effect of increased weight loading in subjects with mild obesity (BMI 30–34.9 kg/m^2^) and further studies should, therefore, also evaluate severely obese (BMI ≥ 35 kg/m^2^), overweight (BMI 25–29.9 kg/m^2^) and normal weight (BMI ≤ 24.9 kg/m^2^) subjects separately as it is suggested from rodent studies that the gravitostat is more efficient in obese than in normal weight rodents [19].

As the average age of the subjects in the present study was rather high (~50 years), the present findings should be confirmed in younger subjects. It is possible that the effect of the sudden increase in body weight (+10%) in the present study differs from the effect of the gradual increase in body weight during normal weight gain. Therefore, future studies should evaluate the effect of slowly increased artificial weight loading.

In conclusion, increased weight loading reduces body weight and fat mass in obese subjects in a similar way as previously shown in obese rodents. These findings demonstrate that there is a weight loading dependent homeostatic regulation of body weight, supporting the gravitostat hypothesis also in humans.

## Author contributions

The design of the study was performed in collaboration between CO, EG, PAJ and JOJ. CO, EG, PAJ and JOJ take primary responsibility for the data and for the fidelity of the study to the protocol. CO and JOJ wrote the first draft of the manuscript. JB and CL were responsible for the calculation of the food intake data. VP, EG and DH was responsible for the serum analyses and the establishment of a validated database of all primary data. All the authors contributed to subsequent drafts of the manuscript and made the decision to submit the manuscript for publication.

## Appendix Supplementary materials

Image, application 1

Image, application 2
