# Supplementary material for: Increased weight loading reduces body weight and body fat in obese subjects – A proof of concept randomized clinical trial
Source: eClinicalMedicine. 2020 Apr 30;22:100338. doi: 10.1016/j.eclinm.2020.100338 (PMC7264953; doi:10.1016/j.eclinm.2020.100338)
Supplement: Supplementary file 1 [file mmc1.docx]

# Supplementary Tables.

**Table S1. Medical history and medications at baseline.**

**Low load High load**  **(N=37) (N=35) p-value**

**Medical history at baseline, n (%)**

Orthopaedic surgery 4 (10.8) 4 (11.4) NS

Hypertension 4 (10.8) 5 (14.3) NS

Arthrosis 3 (8.1) 1 (2.9) NS

Appendectomy 4 (10.8) 1 (2.9) NS

Allergy 9 (24.3) 16 (45.7) NS

Hypothyreosis 6 (16.2) 4 (11.4) NS

Depression 6 (16.2) 5 (14.3) NS

Asthma/COPD 3 (8.1) 3 (8.6) NS

Migraine 0 (0) 2 (5.7) NS

Hypercholesterolemia 0 (0) 2 (5.7) NS

Stroke/thrombosis 0 (0) 2 (5.7) NS

Back pain/disc hernia 5 (13.5) 2 (5.7) NS

Ovarian surgery/hysterectomy/conisation 8 (21.6) 4 (11.4) NS

Wrist fracture 3 (8.1) 0 (0) NS

Meniscus injury 3 (8.1) 0 (0) NS

Tonsillectomy/mandibular/pharyngeal surgery 3 (8.1) 0 (0) NS

Irritable bowel syndrome 1 (2.7) 0 (0) NS

Ligament injury/fibromyalgia 0 (0) 4 (11.4) NS

Diverticulitis/gastroenteritis 2 (5.4) 0 (0) NS

Cholecystitis/cholecystectomy 3 (8.1) 3 (8.6) NS

Eye surgery 1 (2.7) 0 (0) NS

Hernia/abdominal surgery 3 (8.1) 4 (11.4) NS

Psoriasis/onychomycosis 2 (5.4) 2 (5.7) NS

Gastro esophageal reflux 1 (2.7) 0 (0) NS

Type 2 diabetes 0 (0) 2 (5.7) NS

ADHD 1 (2.7) 0 (0) NS

Mb Meniere 0 (0) 1 (2.9) NS

WPW-syndrome 0 (0) 1 (2.9) NS

Bell’s palsy 1 (2.7) 0 (0) NS

Anemia/folate deficiency 0 (0) 2 (5.7) NS

**Low load High load p-value**  (**N=37) (N=35)**

**Medications at baseline, n (%)**

*Antihypertensive medication* 5 (13.5) 7 (20.0) NS

Candesartan 1 (2.7) 0 (0) NS

Felodipine 0 (0) 1 (2.9) NS

Enalapril 2 (5.4) 1 (2.9) NS

Amlodipine 2 (5.4) 1 (2.9) NS

Losartan 0 (0) 3 (8.6) NS Hydrochlorothiazide 0 (0) 1 (2.9) NS

*Analgetics* 2 (5.4) 3 (8.6) NS

Paracetamol 1 (2.7) 1 (2.9) NS

Clorzoxazon 1 (2.7) 1 (2.9) NS

Tramadol 0 (0) 1 (2.9) NS

*Non-steroid anti-inflammatory drugs* 2 (5.4) 1 (2.9) NS

Ibuprofen 1 (2.7) 0 (0) NS

Diclofenac 1 (2.7) 0 (0) NS

Naproxen 0 (0) 1 (2.9) NS

*Supplements/health food/minerals* 19 (51.4) 14 (40.0) NS

Omega-3 fatty acids 2 (5.4) 4 (11.4) NS

Iron substitution 2 (5.4) 0 (0) NS

Magnesium 2 (5.4) 4 (11.4) NS

Varia 13 (35.1) 6 (17.1) NS

*Statins* 0 (0) 2 (5.7) NS

Atorvastatin 0 (0) 1 (2.9) NS

Rosuvastatin 0 (0) 1 (2.9) NS

*Lowering of stomach acid* 2 (5.4) 1 (2.9) NS

Omeprazole 2 (5.4) 1 (2.9) NS

*Antihistamine medication* 1 (2.7) 3 (8.6) NS Desloratadine 0 (0) 2 (5.7) NS

Prometazine 1 (2.7) 0 (0) NS Cromoglicate 0 (0) 1 (2.9) NS

*Glucocorticoids* 1 (2.7) 1 (2.9) NS

Budesonide 1 (2.7) 1 (2.9) NS

*Antidepressant medication* 6 (18.9) 5 (14.3) NS

Citalopram 0 (0) 3 (8.6) NS

Sertraline 4 (10.8) 1 (2.9) NS

Duloxetine 0 (0) 1 (2.9) NS

Lamotrigine 1 (2.7) 0 (0) NS

Agomelatine 1 (2.7) 0 (0) NS

*Sedative-hypnotics* 0 (0) 1 (2.9) NS

Zopiclone 0 (0) 1 (2.9) NS

*Thyroid hormone substitution* 6 (16.2) 4 (11.4) NS

Levothyroxine 6 (16.2) 4 (11.4) NS

*Vitamins* 15 (40.5) 11 (31.4) NS

D-vitamin 7 (18.9) 5 (14.3) NS

Multivitamin 4 (10.8) 3 (8.6) NS

B-vitamin 2 (5.4) 2 (5.7) NS

C-vitamin 2 (5.4) 0 (0) NS

Folate 0 (0) 1 (2.9) NS

*Beta-2-agonists/acetyl cysteine* 3 (8.1) 4 (11.4) NS

Salbutamol 0 (0) 3 (8.6) NS

Terbutaline 2 (5.4) 1 (2.9) NS

Acetyl cysteine 1 (2.7) 0 (0) NS

*Anti-diabetic medication* 0 (0) 1 (2.9) NS

Metformin 0 (0) 1 (2.9) NS

*Hormones* 3 (8.1) 1 (2.9) NS

Gestagens 3 (8.1) 1 (2.9) NS

Progesterone 0 (0) 1 (2.9) NS

*Anti-constipation* 2 (5.4) 1 (2.9) NS Karayagum 2 (5.4) 1 (2.9) NS

*Other medications* 3 (8.1) 3 (8.6) NS

Dexamphetamine 1 (2.7) 0 (0) NS

Allopurinol 1 (2.7) 0 (0) NS

Sulfasalazine 0 (0) 1 (2.9) NS

Terbenafin 0 (0) 1 (2.9) NS

Sildenafil 0 (0) 1 (2.9) NS

Bupropion 1 (2.7) 0 (0) NS

Medical history and medications at baseline are presented as number of subjects together with percentage for all randomized subjects. NS denotes not significant.

**Supplemental Table 2 Per protocol analyses (PPA) of the relative and absolute changes in the primary and secondary outcomes**

|  | **Within group comparison** | |  |  |
| --- | --- | --- | --- | --- |
|  | **Low Load (N=35)** | **High load (N=32)** | **Difference**  **between groups** | **P ANCOVA** |
| ***Primary outcome*** |  |  |  |  |
| *Relative change* |  |  |  |  |
| Body weight (%) | -0.33 (-0.73 to 0.07) | -1.67 (-2.09 to -1.24)*** | -1.34 (-1.94 to -0.74) | 3.8E-05 |
|  |  |  |  |  |
| ***Secondary outcomes*** |  |  |  |  |
| *Relative change* |  |  |  |  |
| Fat mass (%) | -0.84(-2.55 to 0.85) | -4.96 (-6.75 to -3.18)*** | -4.12 (-6.66 to -1.58) | 1.9E-03 |
| Fat free mass (%) | -0.04 (-1.37 to 1.26) | 0.51 (-0.85 to 1.87) | 0.55 (-1.39 to 2.48) | 0.57 |
| Fat percent (%) | -0.52 (-2.27 to 1.19) | -3.35 (-5.14 to -1.55)*** | -2.83 (-5.38 to -0.28) | 0.030 |
|  |  |  |  |  |
| *Absolute change* |  |  |  |  |
| Body weight (kg) | -0.32 (-0.68 to 0.04) | -1.59 (-1.98 to -1.21)*** | -1.27 (-1.82 to -0.73) | 1.6E-05 |
| Fat mass (kg) | -0.24 (-0.85 to 0.37) | -1.78 (-2.42 to -1.14)*** | -1.54 (-2.45 to -0.63) | 1.2E-03 |
| Fat free mass (kg) | -0.08 (-0.74 to 0.59) | 0.19 (-0.51 to 0.89) | 0.26 (-0.73 to 1.26) | 0.60 |
| Fat percent (%) | -0.17 (-0.86 to 0.51) | -1.31 (-2.02 to -0.59)*** | -1.14 (-2.16 to -0.12) | 0.030 |
|  |  |  |  |  |

The primary outcome was the relative change after 3 weeks in body weight. Results are presented as least square means with 95% confidence intervals. Adjustments for age, sex, baseline BMI, Vest exposure (h) and standing % with vest were performed using analysis of covariance (ANCOVA) for comparisons between groups. *** = p<0.001 ** = p<0.01 for within group comparison (week 3 vs baseline) using Wilcoxon signed rank-sum test.

**Supplemental Table 3 Sex stratified analyses of the relative and absolute change in body weight for all study subjects who completed the study.**

|  | **Within group comparison** | |  |  |
| --- | --- | --- | --- | --- |
|  | **Low Load** | **High load** | **Difference**  **between groups** | **P ANCOVA** |
|  |  |  |  |  |
| *Women* | (N=30) | (N=23) |  |  |
| Relative change (%) | -0.17 (-0.63 to 0.30) | -1.66 (-2.19 to -1.13) | -1.49 (-2.22 to -0.77) | 1.4E-04 |
| Absolute change (kg) | -0.12 (-0.53 to 0.29) | -1.48 (-1.95 to -1.00) | -1.36 (-2.00 to -0.71) | 1.1E-04 |
|  |  |  |  |  |
| *Men* | (N=6) | (N=10) |  |  |
| Relative change (%) | -0.87 (-1.58 to -0.16) | -1.84 (-2.36 to -1.31) | -0.97 (-1.92 to -0.01) | 0.048 |
| Absolute change (kg) | -0.91 (-1.68 to -0.15) | -2.08 (-2.64 to -1.52) | -1.17 (-2.19 to -0.14) | 0.029 |

The primary outcome was the relative change after 3 weeks in body weight. Results are presented as least square means with 95% confidence intervals. Adjustments for age, baseline BMI, Vest exposure (h) and standing % with vest were performed using analysis of covariance (ANCOVA) for comparisons between groups

**Supplemental Table 4 Analyses of the relative changes in serum parameters and food intake for all subjects who completed the study**

|  | **Within group comparison** | |  |  |
| --- | --- | --- | --- | --- |
|  | **Low Load** | **High load** | **Difference**  **between groups** | **P ANCOVA** |
|  |  |  |  |  |
| *Serum/plasma* | (N=34) | (N=33) |  |  |
| Total Cholesterol (%) | 2.21 (-1.01 to 5.43) | -0.69 (-3.96 to 2.58) | -2.90 (-7.61 to 1.81) | 0.22 |
| HDL Cholesterol (%) | 1.94 (-1.13 to 5.00) | -0.91 (-4.03 to 2.20) | -2.85 (-7.33 to 1.64) | 0.21 |
| LDL Cholesterol (%) | 3.99 (-0.32 to 8.30) | -3.86 (-8.23 to 0.52) | **-7.84 (-14.15 to -1.54)** | **0.016** |
| Triglycerides (%) | -0.33 (-10.82 to 10.15) | 9.72 (-1.11 to 20.54) | 10.05 (-5.43 to 25.53) | 0.20 |
| Leptin (%) | -6.37 (-14.98 to 2.24) | **-13.59 (-22.33 to -4.84)**** | -7.21 (-19.81 to 5.38) | 0.26 |
| Adiponectin (%) | 5.32 (-1.55 to 12.19) | 3.90 (-3.08 to 10.88) | -1.42 (-11.47 to 8.63) | 0.78 |
| Insulin (%) | -11.55 (-27.56 to 4.46) | 5.49 (-10.77 to 21.76) | 17.04 (-6.38 to 40.47) | 0.15 |
| HOMA index (%) | -9.65 (-26.46 to 7.17) | 6.20 (-10.88 to 23.29) | 15.85 (-8.75 to 40.45) | 0.20 |
|  |  |  |  |  |
|  | (N=36) | (N=33) |  |  |
| Glucose (%) | 1.30 (-1.93 to 4.52) | -0.93 (-4.31 to 2.45) | -2.23 (-7.04 to 2.59) | 0.36 |
|  |  |  |  |  |
|  |  |  |  |  |
| *Food intake* | (N=31) | (N=32) |  |  |
| Week 1 (%) | -2.01 (-13.43 to 9.41) | 1.47 (-9.75 to 12.70) | 3.48 (-13.07 to 20.04) | 0.67 |
|  |  |  |  |  |
|  | (N=34) | (N=32) |  |  |
| Week 2 (%) | 15.47 (-3.92 to 34.86) | 2.88 (-17.14 to 22.90) | -12.59 (-41.20 to 16.02) | 0.38 |
|  |  |  |  |  |
|  | (N=32) | (N=32) |  |  |
| Week 3 (%) | 2.67 (-7.59 to 12.94) | -3.21 (-13.47 to 7.05) | -5.88 (-20.74 to 8.97) | 0.43 |

The relative changes in percent for 3 weeks compared with baseline are given for serum parameters while the relative changes after 1, 2 and 3 weeks vs baseline are given for food intake. Results are presented as least square means with 95% confidence intervals. Adjustments for age, sex, baseline BMI, Vest exposure (h) and standing % with vest were performed using analysis of covariance (ANCOVA) for comparisons between groups. ** = p<0.01 for within group comparison (week 3 vs baseline) using Wilcoxon signed rank-sum test.
