# Supplementary material for: Increased weight loading reduces body weight and body fat in obese subjects – A proof of concept randomized clinical trial
Source: eClinicalMedicine. 2020 Apr 30;22:100338. doi: 10.1016/j.eclinm.2020.100338 (PMC7264953; doi:10.1016/j.eclinm.2020.100338)
Supplement: Supplementary file 2 [file mmc2.pdf]

This supplement contains the following items:

Protocol:

- Original protocol (Pages 2-21)
- Updated protocol (Pages 22-41)

Statistical analysis plan (SAP)

- Original SAP (Page 42),
- Updated SAP (Page 43)

INKOM
2018 -07- 05
Etikprövningsnämnden i
Göteborg

652-18

Original

Bilaga 2

Clinical Study Protocol

Effect of different weight vests on body weight in obese individuals

**Study Code:**	Not applicable
**EudraCT Number:**	Not applicable
**Version Number:**	1.01
**Date:**	2018-04-30
**Investigators:**	Per-Anders Jansson, MD, PhD, Professor<br>Senior consultant at the Clinical Trial Centre,<br>Principle Investigator<br><br>John-Olov Jansson, MD, PhD, Professor<br><br>Claes Ohlsson, MD, PhD, Professor<br><br>Edwin Gidestrand, MD
**Sponsor:**	Per-Anders Jansson, MD, PhD, Professor
**Monitor:**	Not applicable

Original

Synopsis

Title: Effect of different weight vests on body weight in obese individuals

Swedish Title: Effekten av viktsvästar på kroppsvikt hos överviktiga individer

Study period: September 2018 – December 2019 including administrative tasks

Study site: The study will be performed at the Clinical Trial Center at Gothia Forum, Sahlgrenska University Hospital, Gothenburg, Sweden

Investigators:
Per-Anders Jansson, MD, PhD, Professor and Senior Consultant at Clinical Trial Center, Sahlgrenska University hospital, Principal Investigator.
John-Olov Jansson, MD, PhD, Professor at Institute of Neuroscience and Physiology
Claes Ohlsson, MD, PhD, Professor at Institute of Medicine, Centre for Bone and Arthritis Research
Edwin Gidestrand, MD, Sahlgrenska University hospital

Background and study design: Obesity related ailments, such as cardiovascular diseases (CVD) and metabolic disorders are major causes of death in the Western World. The proposed research may result in improved prevention, diagnosis and treatments of obesity and obesity-related disorders. Recently published data show that if a weight is carried by a rodent, this animal will lose body weight and gain an improved glucose control.

We aim to confirm these findings in a human model. We plan to let obese subjects carry weight vests and monitor their change in body weight. We will also measure appetite, physical activity and insulin sensitivity to further examine the potential beneficial effects of loading. Blood sampling will be performed to investigate the mechanism of action.

Objectives: The primary and secondary objectives are detailed in Table 1.

Table 1.

**Primary Objective:**	**Primary Variables/Measures:**
To determine if wearing a weight vest with 11 % of body weight for 8 hours/day for 3 weeks decreases body weight in obese individuals.	Change in body weight (in % of baseline body weight) between three weeks and baseline.
**Secondary Objectives:**	**Secondary Variables/Measures:**
To determine if wearing a weight vest with 11 % of body weight for 8 hours/day for 3 weeks affects fat mass, bone mass, fat free mass and water mass.	Change in fat mass (%), fat free mass (%) between three weeks and baseline measured by bioelectrical impedance analysis.

Original

To determine if wearing a weight vest with 11 % of body weight for 8 hours for 3 weeks affects serum concentrations of glucose, insulin, HDL, LDL, TAG, FGF21, osteocalcin, FGF23, FGF15, sclerostin, lipocalin2, leptin, ghrelin, GLP-1, GLP-2, CCK, (3-36)PYY, glucagon, renin, testosterone, estrogens, methoxynorepinephrine, Na, K, Cl, Ca and creatinine.	Change in plasma concentrations of glucose, insulin, HDL, LDL, TAG, FGF21, osteocalcin, FGF23, FGF15, sclerostin, lipocalin2, leptin, ghrelin, GLP-1, GLP-2, CCK, (3-36)PYY, glucagon, renin, testosterone, estrogens, methoxynorepinephrine, Na, K, Cl, Ca and creatinine during the study days compared to baseline
To determine if wearing a weight vest with 11 % of body weight for 8 hours for 3 weeks affects food intake and appetite.	Reported calorie intake (kcal) and level of hunger each study week through the validated pol MiniMealQ.
To determine if wearing a weight vest with 11 % of body weight for 8 hours for 3 weeks affects physical activity.	Pedometers will be used to measure the subject's activity each day (kcal, walking distance, steps taken).

Statistics: We aim to include at least 40 subjects in each treatment group, resulting in 80 % power to discover a difference in body weight change of ≥ 1.6 %. Student's T-test in SPSS Statistics will be used to compare the two groups.

Contact: Per-Anders Jansson: per-anders.jansson@wlab.gu.se
John-Olov Jansson: joj@neuro.gu.se
Claes Ohlsson: claes.ohlsson@medic.gu.se
Edwin Gidestrand: edwin.gidestrand@vgregion.se

Table of Contents

1. Introduction 6
1.1. Background 6
1.2. Rationale for conducting this study 7
1.3. Risk/Benefit evaluation 8
2. Study Objectives and Endpoints 8
2.1. Primary objective 8
2.2. Secondary objective 8
3. Study Design and Procedures 8
3.1. Overall study design and procedure protocol 8
3.2. Rationale for study design 12
4. Study Population 12
4.1. Inclusion criteria 12
4.2. Exclusion criteria 13
4.3. Restrictions 13
4.4. Subject enrolment and randomization 14
4.5. Discontinuation and withdrawal of subjects 14
4.5.1. Premature termination of the study 14
5. Study Treatments 14
6. Study Measurements and Variables 14
6.1. Variables 14
6.2. Biological sampling procedures 15
6.2.1. Handling, storage and destruction of biological samples 15
6.2.2. Total volume of blood per patient 16
7. Safety 16
8. Statistics 16
8.1. Sample size calculation 16
8.2. Statistical analysis 17
9. Data Management 17
9.1. Recording of data 17
9.1.1. Source data 17
9.2. Data storage and management 17
10. Quality Control and Quality Assurance 18
10.1. Monitoring 18
10.2. Audits and inspections 18
11. Ethics 18
11.1. Ethics committee 18
11.2. Informed consent 18
11.3. Subject data protection 19
11.4. Insurances and compensation 19
12. Protocol Deviations and Amendments 19
13. Report and publications 19
14. Study Timetable 19
14.1. Definition of “End of study” 19
15. List of References 19

Original

List of Abbreviations

B	Blood
BIA	Bioelectrical Impedance Analysis
BMI	Body Mass Index
P	Plasma
S	Serum

Original

1. INTRODUCTION

1.1. Background

According to the latest statistics published by the WHO, more than 1.9 billion adults are overweight, and at least 600 million of them are clinically obese. Worldwide obesity has more than doubled since 1980. Obesity related ailments, such as cardiovascular diseases (CVD) and metabolic disorders are major causes of death in the Western World. The proposed research may result in improved prevention, diagnosis and treatments of obesity and obesity-related disorders. (1)

Epidemiologic studies demonstrate that subjects spending much time sitting have increased risk of obesity, diabetes, and cardiovascular diseases. There is even epidemiologic evidence for an association between sitting time and overall mortality. (2) The mechanism for the anti-obesity effect of standing is essentially unknown. Recently published data by us show that if a weight is carried by a rodent, this animal will lose body weight and get improved glucose metabolism (3).

A fundamental principle of biology is that internal body functions can be kept relatively constant by feedback regulation. (4) Before our publication (3), there only existed one known mediator of this crucial information to the brain about the energy content of fat depots: leptin discovered by Friedman almost twenty five years ago. (5, 6) The importance of leptin as an afferent mediator is illustrated by the profound increase in body fat in animals and humans lacking endogenous leptin, and the reversal of this effect by leptin replacement. (7, 8)

These studies demonstrate that leptin is necessary for fat mass homeostasis. However, most patients with obesity have high endogenous leptin levels and do not respond to leptin treatment, indicating that leptin under these circumstances is not sufficient to suppress body fat mass. This has been referred to as leptin resistance. (7) Contrary to leptin, the body weight loading mechanism, known as the "gravitostat", seem to be present in obese subjects; it even seems to be more effective in obese subjects. Additionally, the gravitostat is regulated by increased loading of the osteocytes which is a completely new approach to obesity (**Figure 1**). (3)

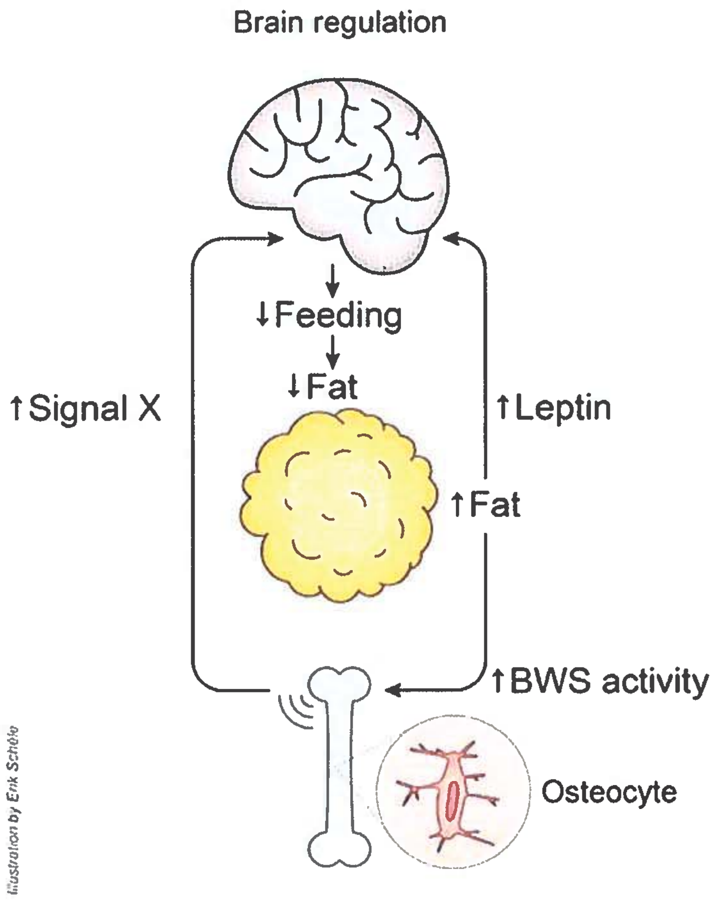

Figure 2. This figure illustrates the two known homeostatic mechanisms of fat regulation: the leptin system and the "gravitostat". Leptin is produced in relation to amount of fat mass and decreases appetite via effects on the brain, possibly the hypothalamus. Leptin is ineffective in obese individuals. Osteocytes decreases food intake when exposed to increased weight. This recently discovered system is known as the "gravitostat" and is present in both normal weight and obese rodents. The appetite decreasing signal that is released from the osteocytes is not yet fully elucidated.

1.2. Rationale for conducting this study

We hypothesized that there is a homeostatic regulation of body weight. Results from experiments based on this hypothesis demonstrate that increased loading of rodents reversibly decreases the biological body weight via reduced food intake. Importantly, loading relieves diet induced obesity and improves glucose tolerance. This novel homeostat for body weight regulates body fat mass independently of fat-derived leptin, revealing two independent negative feed-back systems for fat mass regulation (3). We now want to investigate if there is a similar homeostatic mechanism in action for regulation of body weight in humans.

1.3. Risk/Benefit evaluation

In general this is a study with few risks. The weight vests used in this experiment are in daily use by thousands of people during exercise. During previous experiments performed by our group on approximately 70 medical students no more than one of the students experienced an adverse event. This student experienced mild dizziness which subsided within a few minutes after removing the weight vest and sitting down. However, our previous experiments were only performed for one day on study subjects with normal BMI and for a maximum of three days.

To minimize the risks in our future study we have decided to include only healthy research subjects consuming no or few medications. We will also have medically trained personnel, such as a junior physician and experienced nurses available during the study and perform it at the Sahlgrenska University Hospital. Study subjects are allowed to withdraw their participation in the study at any time.

Blood sampling can be experienced as somewhat uncomfortable by a few individuals. However it is generally free from complications. In some subjects, there may be small local bruising or inflammation.

In summary we believe the risks to be very low compared to the potential of exploring a new physiological mechanism, a mechanism with potentially important applications such as new obesity treatment. There is a great need of better treatment options for obesity.

2. STUDY OBJECTIVES AND ENDPOINTS

2.1. Primary objective

To determine if wearing a weight vest with 11 % of an individual's body weight for 3 weeks decreases body weight in obese study subjects.

2.2. Secondary objective

To determine if wearing a weight vest with 11 % of an individual's body weight for 3 weeks affects fat mass, bone mass, muscle mass, water mass, fat free mass, physical activity and food intake in obese study subjects.

To determine if wearing a weight vest with 11 % of an individual's body weight for 3 weeks affects plasma concentrations of glucose, insulin, HDL, LDL, TAG, FGF21, osteocalcin, FGF23, FGF15, sclerostin, lipocalin-2, leptin, ghrelin, GLP-1, GLP-2, CCK, (3-36)PYY, glucagon, renin, methoxynorepinephrine, Na, K, Cl, Ca and creatinine.

3. STUDY DESIGN AND PROCEDURES

3.1. Overall study design and procedure protocol

Original

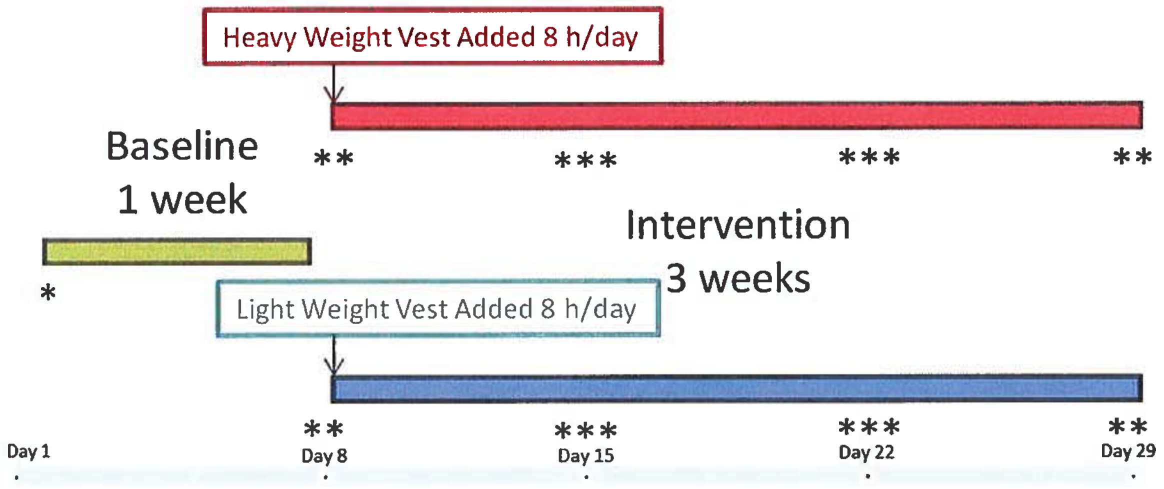

*Informed consent will be collected on day 1.

**Blood sampling and impedance analysis performed on day 8 and 29.

***Telephone contact will be performed on day 15 and 22.

Figure 2. This figure shows the principles of the study. The green line illustrates the first week of the study. Study subjects will have one week to properly learn to count calories and use the pedometers. The red line illustrates the heavy vest group. This group will wear a weight vest with 11 % of body weight for 8 hours each day during three weeks. The blue line illustrates the control group. This group will wear a light weight vest weighing 1 % of body weight for 8 hours each day during three weeks. Calorie intake and physical activity will be measured every study day. *Informing the subject and collecting written consent will be performed on study day 1. **Blood sampling, impedance analysis and contact with medical personnel will be performed on study day 8and 29. ***Telephone contact with subjects will be performed on study day 15 and 22. This is to prevent mistakes and minimize the loss to follow-up.

The principles of the study are shown in **Figure 2**. The aim of the study is to investigate the effect of adding synthetic weights to obese individuals. The weights will be added as a weight vest with 11 % of the individual's body weight and will be worn for three weeks. Half of the study subjects will be given a weight vest with 11 % of body weight and half of the study subjects will be given a light weight vest. The light weight vest consists of a weight vest with most of the weights removed. The weight of the light vest will be 1 % of body weight giving a difference of 10 % between the groups.

Body weight, fat mass, bone mass, water mass and fat free mass will be measured before and after the intervention by medical personnel to determine the effect of added synthetic weights on obese subject. This will be measured using bioelectrical impedance analysis (BIA) (MC-180MA, Tanita). The scales will be calibrated every week during the study to make certain reliable measurements.

Pedometers will be used to measure the subject's activity each study day. We will also encourage the study participants to report food intake, choice of food and level of hunger each week. This will be done using the validated poll MiniMealQ(9). A pedometer will also be attached to the vests to check the daily use of the vests. The study persons will be informed about this arrangement.

Fasting blood sampling will be performed before and after the intervention to further determine the effects of adding synthetic weights to obese individuals but also to elucidate the mechanism behind the “gravitostat”. Blood glucose levels will be measured immediately. Blood samples, except blood glucose, will be analyzed either continuous or after all study subjects have been enrolled depending on human resources.

Study subjects will be recruited by advertising in the local newspaper. Study subjects will be informed about hypothesis that people carrying a heavy or light weight may differ in weight loss. At this point it is not known if the heavy weight vest or the light vest is causing more weight loss, as no one has performed similar studies on humans.

Study subjects will have one week to familiarize themselves with the equipment used in the study before the intervention starts. This is to minimize human errors during the intervention and ensure a high level of compliance. The data collected during this week will be used as baseline.

Day 1 – enrollment

The first 100 persons to contact the researchers will be enrolled in the study. In order to be enrolled a study subject also need to meet all the inclusion criteria and not meet any of the exclusion criteria. The study subjects will be entitled to a private meeting with one of the researchers. Study subjects will receive information about the study plan both orally and written during this meeting.

Consumption of more than one liter of wine (11 %) or alcohol equivalent during a study week will not be accepted. Drastic change in lifestyle before or during the study will not be accepted. Any study subject failing to comply with this restriction will instantly be removed from the study.

Education and detailed planning of the study subjects will be performed after written consent is obtained from the study subject. Body weight, fat mass, bone mass, water mass and fat free mass will be measured using BIA. It is unlikely that bone mass and water mass will be affected during the short time of this study but these values are easily obtained during the measurements. We will also measure blood pressure, height in order to calculate BMI and collect each subject’s age and gender.

This meeting will end with a blood sample to screen for common diseases that may distort the results and increase the risk of adverse effects. We will measure the following substances in blood: hemoglobin, white blood cells, trombocytes, sodium, potassium, creatinine, ASAT, ALAT, HbA1c, C-reactive protein (CRP), T4, TSH. Also, we will use urine dipsticks to further screen for diseases. We will measure the following substances in urine: nitrite, white blood cells, red blood cells, glucose, creatinine and albumin. Only subjects were all above substances are within normal levels will be included in the study.

Study subject will be able to contact the researchers by e-mail or mobile phone any time during the study if questions arise.

Day 8 – intervention starts

Study subjects will be entitled to another appointment with the researchers and the nurse from Gothia Forum one week after the enrollment. Body weight, fat mass, bone mass, fat free mass and water mass will be measured during this meeting using BIA.

Fasting blood sampling by an experienced nurse from Gothia Forum will be performed when the researcher believe that the study subject is fully educated and ready to be enrolled in the study. A maximum of 25 ml will be collected at each blood sampling. Plasma levels of glucose will be measured instantly in capillary blood, and thus not be dependent on successful venous blood sampling. Venous blood samples for other measurement than glucose will be centrifuged by the researchers or personnel from Gothia Forum to obtain serum or EDTA plasma samples. Serum and plasma samples may be stored at a temperature of -80°C until analysis. Study subjects can request to have their blood samples destroyed at any time.

The intervention will start directly after blood sampling. Subjects will earn a randomized three character code to be used throughout the study; this will be noted in a digital only randomization list. Study subjects will obtain either a weight vest with 11 % of body weight or a light vest weighing 1 % of body weight. The vest is to be worn 8 hours each day for the following 3 weeks. The vest is only to be used during awake hours.

Day 15

Study subjects will be entitled to another appointment with the researchers two weeks after the enrollment, this appointment may be performed by telephone if the subject so agree. Any problems with compliance will be discussed. Study subjects who deviate more than 20 % from the study protocol will be removed from the study. Study subjects will continue to wear the vests for 8 hours each day.

Day 22

Study subjects will be entitled to another appointment with the researchers three weeks after the enrollment, this appointment may be performed by telephone if the subject so agree. Any problems with compliance will be discussed. Study subjects who deviate more than 20 % from the study protocol will be removed from the study. Study subjects will continue to wear the vests for 8 hours each day.

Day 29 – intervention ends

Study subjects will be entitled to another appointment in the morning before breakfast with the researchers and the nurse from Gothia Forum four weeks after the enrollment. Body weight, fat mass, bone mass, fat free mass and water mass will be measured during this meeting using BIA. Fasting glucose will be measured in capillary blood. Venous blood sampling will be done. Study subjects will have worn the vests for 8 hours the day before, but they will not wear it during the visit the morning of day 29.

The appointment at day 29 may also be performed on "day 30" depending on the study subject's schedule. Nevertheless, last day for the intervention is on study day 29.

Table 2. This table summarizes all the procedures. Blood samples, except blood glucose, will be analyzed either continuous or after all study subjects have been enrolled depending on human resources.

Table 2 **Study plan detailing the procedures each day**

Time point (day)	1	1-8	8	8-15	15	15-22	22	22-29	29
Window	+/- 2h	+/- 2h	+/- 2 days	+/- 2h	+/- 2h	+/- 2h	+/- 2h	+/- 2h	+/- 2 days
Height for BMI	X								

Original

Time point (day)	1	1-8	8	8-15	15	15-22	22	22-29	29
**Window**	+/- 2h	+/- 2h	+/- 2 days	+/- 2h	+/- 2h	+/- 2h	+/- 2h	+/- 2h	+/- 2 days
Body weight	X		X						X
Impedance	X		X						X
Fasting Blood Sample			X						X
Blood Glucose			X						X
Screening blood sample	X								
Intervention starts			X						
Intervention ends									X
Intervention (Heavy Weight Vest or Light Vest 8 h/day)				X		X		X	
Reporting Food Intake		X		X		X		X	
Measuring physical activity		X		X		X		X	

3.2. Rationale for study design

In order to avoid the risk of carry-over effects, we have decided to not use a crossover design. This could have decreased the variation of the measurement values and increased the power of the study. However, we believe there could be a risk of a carry-over effect with a cross over design. As this is a completely new effect in clinical studies, we have no information about the wash-out time needed to avoid this problem.

4. STUDY POPULATION

4.1. Inclusion criteria

For inclusion in the study, subjects must fulfil the following criteria:

1. Obesity as defined by a BMI >30 and ≤35. Fat mass should be above 25 % (10).
2. 18-65 years of age. We will primarily recruit men. Women will only be recruited if we are unable to find 80 eligible male participants.
3. Consent out of free will.

4. Willingness to comply with the study protocol and restrictions of not consuming excessive amounts of alcohol (maximum 1 litre, 11 % or alcohol equivalent for a full week) or using any drugs. Smoking and snuff use is allowed.

5. Normal screening blood- and urine samples. In blood: hemoglobin, white blood cells, trombocytes, sodium, potassium, creatinine, ASAT, ALAT, HbA1c, CRP, T4, TSH. In urine: nitrite, white blood cells, red blood cells, glucose, creatinine and albumin.

6. Signed informed consent.

4.2. Exclusion criteria

Subjects must not enter the study if any of the following are present:

1. Chronic disease that hardens the participation in the study as judged by the investigator.

2. Chronic pain such as pain that is constant and impairs quality of life; for example: severe back, hip and knee pain.

3. Regular consumption of medicine or natural supplements that affect weight, inhibit physical activity or increase the risk of adverse effects as judged by the investigator. The following drugs will not be accepted: β-antagonists, GLP-1-agonists, SGLT2-inhibitors, sulfonylureas, insulin, orlistat, mysimba and bisphosphonates.

4. Gastric by-pass surgery or equivalent.

5. Reduced mobility.

6. Pregnancy: Females of childbearing potential must confirm to use reliable contraception and not suspect to be pregnant. Subjects may be asked to perform a pregnancy test.

7. Change in body weight of 5 kg or greater during the past 3 months or recently started a strict diet. Also, a greater change in body weight than 1.5 kg difference between day 1 and day 8 will not be accepted.

8. Drastic change in lifestyle during the last 3 months; for example a significant change in physical activity or nicotine, alcohol or drug use.

9. Apparent risk of not being able to comply with the study protocol for any reason as judged by the investigator.

Reasons for exclusion of participants will be reported in the publication.

4.3. Restrictions

The study subjects will not be allowed to remove the vests for 8 hours upon putting it on. Subjects who deviate more than 20 % from this (i.e. use the vest for less than 6.4 hours during the daily 8 hour vest wearing period) will be excluded from the study.

The study subjects are only allowed to consume a limited amount of alcohol and other drugs during the study. This is in line with the inclusion- and exclusion criteria.

4.4. Subject enrolment and randomization

We will recruit healthy individuals by advertising in a local newspaper. We will only enroll healthy people who do not know the hypothesis of the study. Each study subject will be given a randomized code to use for the study. It will then be decided by dice throw if a study subject is to be included in the group wearing heavy weight vests or light weight vests. Due to the risk of drop-outs we will aim to recruit 50 participants in each group for a total of 100 study subjects. However, 40 participants are needed to receive a power of 80 % to discover a difference in body weight change of ≥ 1.6 %.

The last participants to enroll in the study may be placed in the group with the least number of study subjects by the researchers if a total of 50 study subjects already have been enrolled in one of the groups. Only the study subject and the investigator will be able to connect each study subject with their randomized code. Identification of individual study subjects will only be performed under special circumstances, for example, if a study subject requests removal of his or her study data.

Study subject eligibility will be established before enrolment and randomization. Study subjects will be randomized strictly sequentially, as study subjects are eligible for randomization. If a study subject discontinues from the study, the study subject number will not be reused, and the study subject will not be allowed to re-enter the study.

4.5. Discontinuation and withdrawal of subjects

Subjects are free to discontinue their participation in the study at any time without prejudice to further treatment. The subjects may be withdrawn from the study at the discretion of the investigator due to safety concerns or if judged non-compliant with study procedures. In either case, serious adverse events will be followed up. Other reasons for discontinuing a subject are incorrect enrolment and subjects lost to follow-up.

4.5.1. Premature termination of the study

The research group may decide to stop the trial or part of the trial at any time. If a trial is prematurely terminated or suspended, the investigator should promptly inform the study subjects. Furthermore, the investigator should promptly inform the Ethics Committee and provide a detailed written explanation.

5. STUDY TREATMENTS

No drugs or medicinal treatment are used in this study. However, study subjects are obliged to wear a weight vest with 11 % of body weight. As discussed previously we judge that this is a study with few risks and only mild potential adverse effects.

6. STUDY MEASUREMENTS AND VARIABLES

6.1. Variables

The primary variable to be measured will be the change of body weight in % of the baseline body weight.

The following parameters are measured during the experiment:

1. Bioelectrical impedance analysis measurement at study day 1, 8, and 29.

Body weight will be measured (kg) which will be used to calculate the change in bodyweight (kg) between the measurements. We will also measure the study subject's height in order to calculate changes in BMI.

Bioelectrical impedance analysis measurement will also be used to calculate change in fat mass (kg), bone mass (kg), fat free mass (kg) and water mass (kg).

2. Blood samples at study day 1, 8, and 29.

Measurements by the Sahlgrenska Academy, University of Gothenburg: insulin, HDL, LDL, TAG, FGF21, osteocalcin, FGF23, FGF15, sclerostin, lipocalin2, leptin, ghrelin, GLP-1, GLP-2, CCK, (3-36)PYY, glucagon, testosterone, estrogens, renin and methoxynorepinephrine.

Measurements by Sahlgrenska University hospital (central lab and clinical trial center): plasma concentration of Na, K, Cl, Ca and creatinine. Sahlgrenska University hospital will also measure the day 1 screening blood- and urine samples. This includes the following samples in blood: hemoglobin, white blood cells, trombocytes, sodium, potassium, creatinine, ASAT, ALAT, HbA1c, CRP, T4, TSH. Urine samples will be analyzed using urine dipstick for the following substances: nitrite, white blood cells, red blood cells, glucose, creatinine and albumin.

3. Self-reported food intake. Choice of food, energy (kcal) and hunger will be assessed.

4. Physical activity will be monitored by pedometers. Energy expenditure by physical activity (kcal), and total mobility (walking distance, steps taken) will be assessed.

6.2. Biological sampling procedures

6.2.1. Handling, storage and destruction of biological samples

To determine the mechanism of action and elucidate the beneficial effects of body weight loading we will analyze blood samples. Some tubes may be used for analyzes by Sahlgrenska University hospital central lab and the rest of the tubes will be used for analyzes by the research group on site or at the Institute of Neurology and Physiology. All samples will be impossible to connect to a study subject without knowing their randomization code. Measurements will be performed as quickly as possible and samples will then be destroyed. At any time, a study subject can request destruction of his or her blood samples.

Each venous blood drawing consists of a maximum of 25 ml. Every sample will then be divided in four tubes – each consisting of 5-6 ml for measurements. The tubes used for blood drawings will contain ethylenediaminetetraacetic acid (EDTA) or a SST-gel for the purpose of manipulating coagulation.

Fasting blood glucose levels will be measured directly in capillary blood using glucose strips and a glucometer.

The samples may be stored at the Sahlgrenska Academy before the actual measurements because of practicalities. All samples will be stored anonymized with only the randomization code on it at a temperature of -80°C. This has been reported to the Swedish "health and Social Care Inspectorate" according to regulation 2002:297.

If we experience technical difficulties measuring any of the above parameters some samples may be sent to another EU/EES country for measurements. The samples will of course be anonymized during the full process. The analysis may take place at a cooperative group, the industry or drug manufacturer. The samples will be returned or destroyed upon completed analysis.

6.2.2. Total volume of blood drawn per patient

Each blood sample will consist of approximately 25 ml blood and divided into four tubes. It means that in total a maximum of 75 ml blood will be taken during the study. In comparison, a blood donation consists of up to 500 ml.

7. SAFETY

In general this is a study with few risks. The weight vests used in this experiment are daily used by thousands of people for exercise. During previous experiments on more than 70 medical students only one of the students were affected with an adverse event. This student experienced mild dizziness which subsided within a few minutes after removing the weight vest and sitting down. However, it should be noted that the risks of wearing a weight vest for several weeks in a row are largely unknown.

To minimize the risks of our future study we have decided to include only healthy research subjects not consuming any or few medication. We will also have medical trained personnel available during the study and perform it in close proximity to the Sahlgrenska University Hospital.

Blood sampling can be experienced as somewhat uncomfortable by a few individuals. However it is generally free from complications. Occasionally, there may be small local bruising and local inflammation. We may fail to get venous blood from 5-15% of these obese subjects. They will still be part of the study, and their levels of the primary outcome body weight will be included in the study.

If an adverse event occurs the study subject can abort the study whenever he or she wants to. The medical personnel involved in the study will help the subject to the correct medical care giver practice if needs arises. Adverse events will be reported in the publication.

8. STATISTICS

8.1. Sample size calculation

Based on our extensive data demonstrating that increased loading decreases body weight in mice and rats (3) we will perform a randomized interventional study to determine if increased loading decreases body weight also in humans. Subjects will be randomized (stratified for gender) to treatment with either (i) increased loading by using a Casall HIT weight vest that weighs 11 % of the baseline body weight (=High load-treated) or (ii) essentially unchanged loading by using a vest with much lower weight (1% of baseline body weight, = Low load treated). The experimental subjects will carry the vest for 8 awake hours per day for 3 weeks.

The primary endpoint of this randomized study will be change in body weight expressed as percent of original weight between day 8 and 28, i.e. the days of the intervention. These values will be calculated for all individuals participating in the study. These values will then be compared between the groups of individuals exposed 11% and 1 % extra loading, using student's t-test. We aim to include at least 40

subjects in each treatment group, resulting in 80 % power to discover a difference in body weight change of ≥ 1.6 %. A total of 100 study subjects will be recruited to achieve a minimum of 40 subjects in each group. Inclusion criteria consists of but is not limited to BMI 30-35 kg/m2, stable body weight during the last six months (less than five kg change and less than 1.5 kg measured change during the first week of the study), age 18-65 years and that the individuals are essentially healthy. The full list of inclusion and exclusion criteria can be found at section 4.1 and 4.2. The plan is to primarily include men because it has been shown that their variability in body weight measured at 3-5 week intervals is less than for women (SD 1.0 kg for men, 1.3 kg for women).

Secondary endpoints will include: Fat mass, bone mass, water mass and fat free mass measured with bioimpedance, serum glucose levels, serum leptin levels and possibly also serum levels of the putative body weight regulating endocrine factor released from osteocytes. Potential body weight regulating factors are listed at section 6.1. Furthermore, we will assess food intake (kcal) and physical activity (kcal, walking distance, steps taken).

8.2. Statistical analysis

Student's T-test in SPSS Statistics will be used to compare the two groups. One calculation will be used for each of the endpoints although we only have one primary endpoint. Subjects who have used the weight vest 3 SD less than mean will be excluded from the analysis.

9. DATA MANAGEMENT

9.1. Recording of data

The investigator will ensure that all data collected in the study are recorded in a timely manner according to any instructions provided.

An electronic Case Report Form will be used for data collection. The investigator will ensure that the data are recorded in the Case Report Form as specified in the study protocol and in accordance with the instructions provided. The investigator ensures the accuracy, completeness and timeliness of the data recorded. The investigator will sign the completed Case Report Form. A copy of the completed Case Report Form will be archived at the study site, in this case The Sahlgrenska Academy.

9.1.1. Source data

The following documents comprise the source data and will be recorded in the Case Report Form at the study site:

- An individual procedure protocol which is an excel file consisting of each study subjects reported calorie intake, physical activity and the results of the bioelectrical impedance analysis measurements. Gender, length, inclusion criteria and other specific information for that particular study subjects will also be included in this file.
- The measurement values obtained from Sahlgrenska University hospital central lab.
- The measurement values obtained from the analyses at the Sahlgrenska Academy.
- A copy of the informed consent from each study subject.

9.2. Data storage and management

All data should be recorded, handled and stored in a way that allows its accurate reporting, interpretation and verification. All raw data, a copy of the completed Case Report Form, original protocol with amendments and the final report will be stored for a minimum period of ten years after termination of the trial, in accordance with Swedish regulation/law (Chapter 10, 3 § in LVFS 2011:19).

At the conclusion of the study, the occurrence of any protocol deviations will be determined. After these actions have been completed and the database has been declared to be complete and accurate, it will be locked and available for data analysis.

10. QUALITY CONTROL AND QUALITY ASSURANCE

10.1. Monitoring

The investigators will have close contact to Gothia Forum during the entire study until the publication; this is to ensure that the investigators are appropriately trained and informed about patient written information, good clinical practice and applicable regulatory requirements. The research group may also consult external experts to verify and correct the data analyses if needed.

10.2. Audits and inspections

Authorized representatives of an Ethics Committee may perform audits or inspection at the institution, including source data verification. The purpose of an audit or inspection is to systematically and independently examine all study-related activities and documents, to determine whether these activities were conducted, and data were recorded, analyzed and accurately reported according to the protocol, Good Clinical Practice and any applicable regulatory requirements.

11. ETHICS

The study will be performed in accordance with ethical principles that have their origin in the Declaration of Helsinki and are consistent with Good Clinical Practice and applicable regulatory requirements.

11.1. Ethics committee

The final study protocol, including the final version of the Informed Consent Form and other information given to subjects must be approved or given a favorable opinion in writing by an Ethics Committee as appropriate. The Principal Investigator is responsible for informing the Ethics Committee of any amendment to the protocol, in accordance with local requirements.

11.2. Informed consent

The Principal Investigator will ensure that the subject is given full and adequate oral and written information about the nature, purpose and possible risks and benefits of the study. Subjects must also be notified that they are free to discontinue from the study at any time. The subject should be given the opportunity to ask questions and allowed time to consider the information provided. The subjects will also be able to contact the researchers using phone or e-mail at any time during the study period.

The subject's signed and dated informed consent must be obtained before conducting any procedure specifically for the study.

The original, signed Informed Consent Form must be stored in the Investigator's Study File. A copy of the signed Informed Consent Form must be given to the subject.

If a protocol amendment requires a change to the Informed Consent Form, the Ethics Committee must approve modifications that lead to a revised Informed Consent Form before the revised form is used.

11.3. Subject data protection

The Informed Consent Form will incorporate wording that complies with relevant data protection and privacy legislation. Pursuant to this wording, subjects will authorize the collection, use and disclosure of their study data by the investigators.

The Informed Consent Form will explain that study data will be stored in a computer database, maintaining confidentiality in accordance with national data legislation. All data computer processed by the investigators will be identified by a randomized code.

11.4. Insurances and compensation

The study subjects are insured by "Kammarkollegiet". A compensation of 500 SEK will be payed to each research subject.

12. PROTOCOL DEVIATIONS AND AMENDMENTS

Modifications to the signed protocol are only possible through approved protocol amendments and with the agreement of all responsible persons. Details of non-substantial amendments are to be clearly noted in the amended protocol. In case of a substantial protocol amendment, the concerned Ethics Committee must be informed and should be asked for its approval prior implementation of amended protocol, as to whether a full re-evaluation of the ethical aspects of the study is necessary by the committee. This should be fully documented.

13. REPORT AND PUBLICATIONS

After completion of the study, the results will be analyzed and a study report will be prepared. In addition, upon study completion and finalization of the study report the results of this trial will be either submitted for publication or posted in a publicly accessible database of clinical trial results.

14. STUDY TIMETABLE

The study will take place between Q3 2018 and Q1 2019. All analyzes shall be finished before 2022-12-31. Publications of the results are planned for late 2019, but may not happen until 2022.

15. LIST OF REFERENCES

1. WHO. Obesity and overweight http://www.who.int/mediacentre/factsheets/fs311/en/: WHO; 2016 [updated June 2016]. Available from: http://www.who.int/mediacentre/factsheets/fs311/en/.
2. Katzmarzyk PT, Church TS, Craig CL, Bouchard C. Sitting time and mortality from all causes, cardiovascular disease, and cancer. Medicine & Science in Sports & Exercise. 2009;41(5):998-1005.
3. Jansson J-O, Palsdottir V, Hägg DA, Schéle E, Dickson SL, Anesten F, et al. Body weight homeostat that regulates fat mass independently of leptin in rats and mice. Proceedings of the National Academy of Sciences. 2018;115(2):427-32.

4. Cannon W. The Wisdom of the Body. 312 pp. New York. 1932.
5. Zhang Y, Proenca R, Maffei M, Barone M, Leopold L, Friedman JM. Positional cloning of the mouse obese gene and its human homologue. Nature. 1994;372(6505):425.
6. Friedman J. 20 years of leptin: leptin at 20: an overview. Journal of endocrinology. 2014;223(1):T1-T8.
7. Frederich RC, Hamann A, Anderson S, Löllmann B, Lowell BB, Flier JS. Leptin levels reflect body lipid content in mice: evidence for diet-induced resistance to leptin action. Nature medicine. 1995;1(12):1311.
8. Montague CT, Farooqi IS, Whitehead JP, Soos MA, Rau H, Wareham NJ, et al. Congenital leptin deficiency is associated with severe early-onset obesity in humans. Nature. 1997;387(6636):903.
9. Christensen SE, Möller E, Bonn SE, Ploner A, Wright A, Sjölander A, et al. Two new meal-and web-based interactive food frequency questionnaires: validation of energy and macronutrient intake. Journal of medical Internet research. 2013;15(6).
10. Larsson I, Forslund HB, Lindroos A, Lissner L, Näslund I, Peltonen M, et al. Body composition in the SOS (Swedish Obese Subjects) reference study. International journal of obesity. 2004;28(10):1317.

Bilaga 2

Clinical Study Protocol

Effect of different weight vests on body weight in obese individuals

**Study Code:**	Not applicable
**EudraCT Number:**	Not applicable
**Version Number:**	2.0
**Date:**	2018-09-10

Investigators:

Per-Anders Jansson, MD, PhD, Professor
Senior consultant at Gothia Forum,
Principle Investigator

John-Olov Jansson, MD, PhD, Professor

Claes Ohlsson, MD, PhD, Professor

Edwin Gidestrand, MD

Sponsor:

John-Olov Jansson, MD, PhD, Professor
Sahlgrenska University Hospital, Gothenburg

Monitor: Not applicable

Synopsis

Title: Effect of Different Weight Vests on Body Weight in Obese Individuals

Swedish Title: Effekten av viktvästar på kroppsvikt hos överviktiga individer

Study period: September 2018 – December 2019 including administrative tasks

Study site: The study will be performed at the Clinical Trial Center at Gothia Forum, Sahlgrenska University Hospital, Gothenburg, Sweden

Investigators:
Per-Anders Jansson, MD, PhD, Professor and Senior Consultant at Gothia Forum, Sahlgrenska University hospital, Principal Investigator.
John-Olov Jansson, MD, PhD, Professor at Institute of Neuroscience and Physiology
Claes Ohlsson, MD, PhD, Professor at Institute of Medicine, Centre for Bone and Arthritis Research
Edwin Gidestrand, MD, Sahlgrenska University hospital

Background and study design: Obesity related ailments, such as cardiovascular diseases (CVD) and metabolic disorders are major causes of death in the Western World. The proposed research may result in improved prevention, diagnosis and treatments of obesity and obesity-related disorders. Recently published data show that if a weight is carried by a rodent, this animal will lose body weight and gain an improved glucose control.

We aim to confirm these findings in a human model. We plan to let obese subjects carry weight vests and monitor their change in body weight. We will also measure appetite, physical activity and insulin sensitivity to further examine the potential beneficial effects of loading. Blood sampling will be performed to investigate the mechanism of action.

Objectives: The primary and secondary objectives are detailed in Table 1.

Table 1.

**Primary Objective:**	**Primary Variables/Measures:**
To determine if wearing a weight vest with 11 % of body weight for 8 hours/day for 3 weeks decreases body weight in obese individuals.	Change in body weight (in % of baseline body weight) between three weeks and baseline.
**Secondary Objectives:**	**Secondary Variables/Measures:**
To determine if wearing a weight vest with 11 % of body weight for 8 hours/day for 3 weeks affects fat mass, bone mass, fat free mass and water mass.	Change in fat mass (%), fat free mass (%) between three weeks and baseline measured by bioelectrical impedance analysis.

To determine if wearing a weight vest with 11 % of body weight for 8 hours for 3 weeks affects serum concentrations of glucose, insulin, HDL, LDL, TG, FGF21, osteocalcin, FGF23, FGF15, sclerostin, lipocalin2, leptin, ghrelin, GLP-1, GLP-2, CCK, (3-36)PYY, glucagon, renin, testosterone, estrogens, methoxynorepinephrine, Na, K, Cl, Ca and creatinine.	Change in plasma concentrations of glucose, insulin, HDL, LDL, TG, FGF21, osteocalcin, FGF23, FGF15, sclerostin, lipocalin2, leptin, ghrelin, GLP-1, GLP-2, CCK, (3-36)PYY, glucagon, renin, testosterone, estrogens, methoxynorepinephrine, Na, K, Cl, Ca and creatinine during the study days compared to baseline
To determine if wearing a weight vest with 11 % of body weight for 8 hours for 3 weeks affects food intake and appetite.	Reported calorie intake (kcal) and level of hunger each study week through the validated pol MiniMealQ.
To determine if wearing a weight vest with 11 % of body weight for 8 hours for 3 weeks affects physical activity.	Pedometers will be used to measure the subject's activity each day (kcal, walking distance, steps taken).

Statistics: We aim to include at least 40 subjects in each treatment group, resulting in 80 % power to discover a difference in body weight change of ≥ 1.6 %. Student's T-test in SPSS Statistics will be used to compare the two groups.

Contact: Per-Anders Jansson: per-anders.jansson@medic.gu.se
John-Olov Jansson: joj@neuro.gu.se
Claes Ohlsson: claes.ohlsson@medic.gu.se
Edwin Gidestrand: edwin.gidestrand@vgregion.se

Table of Contents

1. Introduction ... 6
1.1. Background ... 6
1.2. Rationale for conducting this study ... 7
1.3. Risk/Benefit evaluation ... 8
2. Study Objectives and Endpoints ... 8
2.1. Primary objective ... 8
2.2. Secondary objective ... 8
3. Study Design and Procedures ... 8
3.1. Overall study design and procedure protocol ... 8
3.2. Rationale for study design ... 12
4. Study Population ... 12
4.1. Inclusion criteria ... 12
4.2. Exclusion criteria ... 13
4.3. Restrictions ... 13
4.4. Subject enrolment and randomization ... 14
4.5. Discontinuation and withdrawal of subjects ... 14
4.5.1. Premature termination of the study ... 14
5. Study Treatments ... 14
6. Study Measurements and Variables ... 14
6.1. Variables ... 15
6.2. Biological sampling procedures ... 15
6.2.1. Handling, storage and destruction of biological samples ... 15
6.2.2. Total volume of blood per patient ... 16
7. Safety ... 16
8. Statistics ... 16
8.1. Sample size calculation ... 16
8.2. Statistical analysis ... 17
9. Data Management ... 17
9.1. Recording of data ... 17
9.1.1. Source data ... 17
9.2. Data storage and management ... 17
10. Quality Control and Quality Assurance ... 18
10.1. Monitoring ... 18
10.2. Audits and inspections ... 18
11. Ethics ... 18
11.1. Ethics committee ... 18
11.2. Informed consent ... 18
11.3. Subject data protection ... 19
11.4. Insurances and compensation ... 19
12. Protocol Deviations and Amendments ... 19
13. Report and publications ... 19
14. Study Timetable ... 20
15. List of References ... 21

List of Abbreviations

ANOVA	Analysis of Variance
B	Blood
BIA	Bioelectrical Impedance Analysis
BMI	Body Mass Index
P	Plasma
S	Serum

1. INTRODUCTION

1.1. Background

According to the latest statistics published by the WHO, more than 1.9 billion adults are overweight, and at least 600 million of them are clinically obese. Worldwide obesity has more than doubled since 1980. Obesity related ailments, such as cardiovascular diseases (CVD) and metabolic disorders are major causes of death in the Western World. The proposed research may result in improved prevention, diagnosis and treatments of obesity and obesity-related disorders. (1)

Epidemiologic studies demonstrate that subjects spending much time sitting have increased risk of obesity, diabetes, and cardiovascular diseases. There is even epidemiologic evidence for an association between sitting time and overall mortality. (2) The mechanism for the anti-obesity effect of standing is essentially unknown. Recently published data by us show that if a weight is carried by a rodent, this animal will lose body weight and get improved glucose metabolism (3).

A fundamental principle of biology is that internal body functions can be kept relatively constant by feedback regulation. (4) Before our publication (3), there only existed one known mediator of this crucial information to the brain about the energy content of fat depots: leptin discovered by Friedman almost twenty five years ago. (5, 6) The importance of leptin as an afferent mediator is illustrated by the profound increase in body fat in animals and humans lacking endogenous leptin, and the reversal of this effect by leptin replacement. (7, 8)

These studies demonstrate that leptin is necessary for fat mass homeostasis. However, most patients with obesity have high endogenous leptin levels and do not respond to leptin treatment, indicating that leptin under these circumstances is not sufficient to suppress body fat mass. This has been referred to as leptin resistance. (7) Contrary to leptin, the body weight loading mechanism, known as the "gravitostat", seem to be present in obese subjects; it even seems to be more effective in obese subjects. Additionally, the gravitostat is regulated by increased loading of the osteocytes which is a completely new approach to obesity (**Figure 1**). (3)

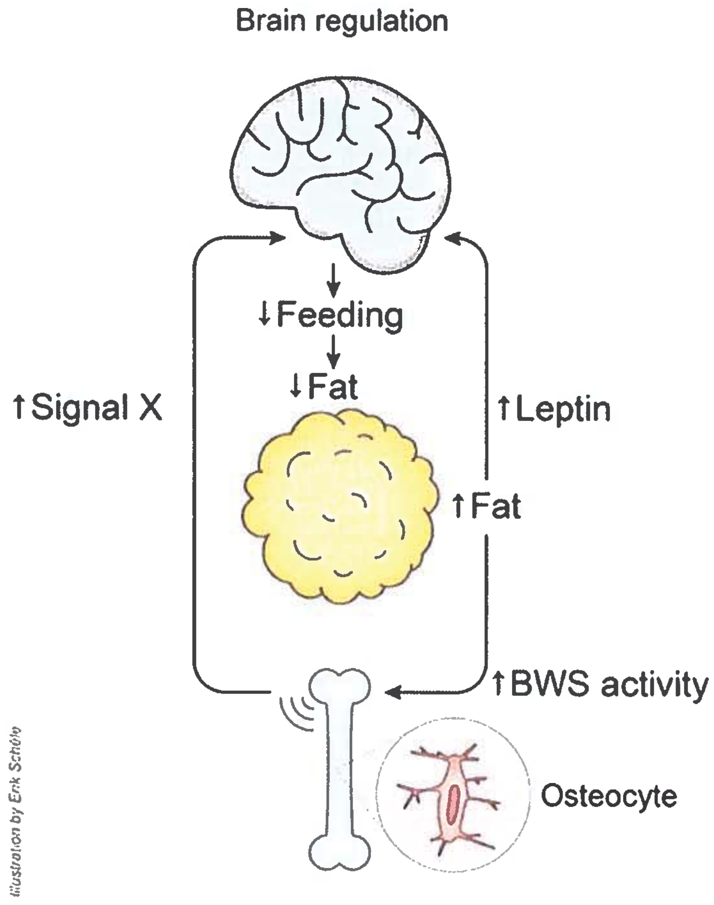

Figure 2. This figure illustrates the two known homeostatic mechanisms of fat regulation: the leptin system and the "gravitostat". Leptin is produced in relation to amount of fat mass and decreases appetite via effects on the brain, possibly the hypothalamus. Leptin is ineffective in obese individuals. Osteocytes decreases food intake when exposed to increased weight. This recently discovered bsystem is known as the "gravitostat" and can be compared to a body weight scale (BWS). It is present in both normal weight and obese rodents. The appetite decreasing signal that is released from the osteocytes is not yet fully elucidated.

1.2. Rationale for conducting this study

We hypothesized that there is a homeostatic regulation of body weight. Results from experiments based on this hypothesis demonstrate that increased loading of rodents reversibly decreases the biological body weight via reduced food intake. Importantly, loading relieves diet induced obesity and improves glucose tolerance. This novel homeostat for body weight regulates body fat mass independently of fat-derived leptin, revealing two independent negative feed-back systems for fat mass regulation (3). We now want to investigate if there is a similar homeostatic mechanism in action for regulation of body weight in humans.

1.3. Risk/Benefit evaluation

In general this is a study with few risks. The weight vests used in this experiment are in daily use by thousands of people during exercise. During previous experiments performed by our group on approximately 70 medical students no more than one of the students experienced an adverse event. This student experienced mild dizziness which subsided within a few minutes after removing the weight vest and sitting down. However, our previous experiments were only performed for one day on study subjects with normal BMI and for a maximum of three days.

To minimize the risks in our future study we have decided to include only healthy research subjects consuming no or few medications. We will also have medically trained personnel, such as a junior physician and experienced nurses available during the study and perform it at the Sahlgrenska University Hospital. Study subjects are allowed to withdraw their participation in the study at any time.

Blood sampling can be experienced as somewhat uncomfortable by a few individuals. However it is generally free from complications. In some subjects, there may be small local bruising or inflammation.

In summary we believe the risks to be very low compared to the potential of exploring a new physiological mechanism, a mechanism with potentially important applications such as new obesity treatment. There is a great need of better treatment options for obesity.

2. STUDY OBJECTIVES AND ENDPOINTS

2.1. Primary objective

To determine if wearing a weight vest with 11 % of an individual's body weight for 3 weeks decreases body weight in obese study subjects.

2.2. Secondary objective

To determine if wearing a weight vest with 11 % of an individual's body weight for 3 weeks affects fat mass, bone mass, muscle mass, water mass, fat free mass, physical activity and food intake in obese study subjects.

To determine if wearing a weight vest with 11 % of an individual's body weight for 3 weeks affects plasma concentrations of glucose, insulin, HDL, LDL, TG, FGF21, osteocalcin, FGF23, FGF15, sclerostin, lipocalin-2, leptin, ghrelin, GLP-1, GLP-2, CCK, (3-36)PYY, glucagon, renin, methoxynorepinephrine, Na, K, Cl, Ca and creatinine.

3. STUDY DESIGN AND PROCEDURES

3.1. Overall study design and procedure protocol

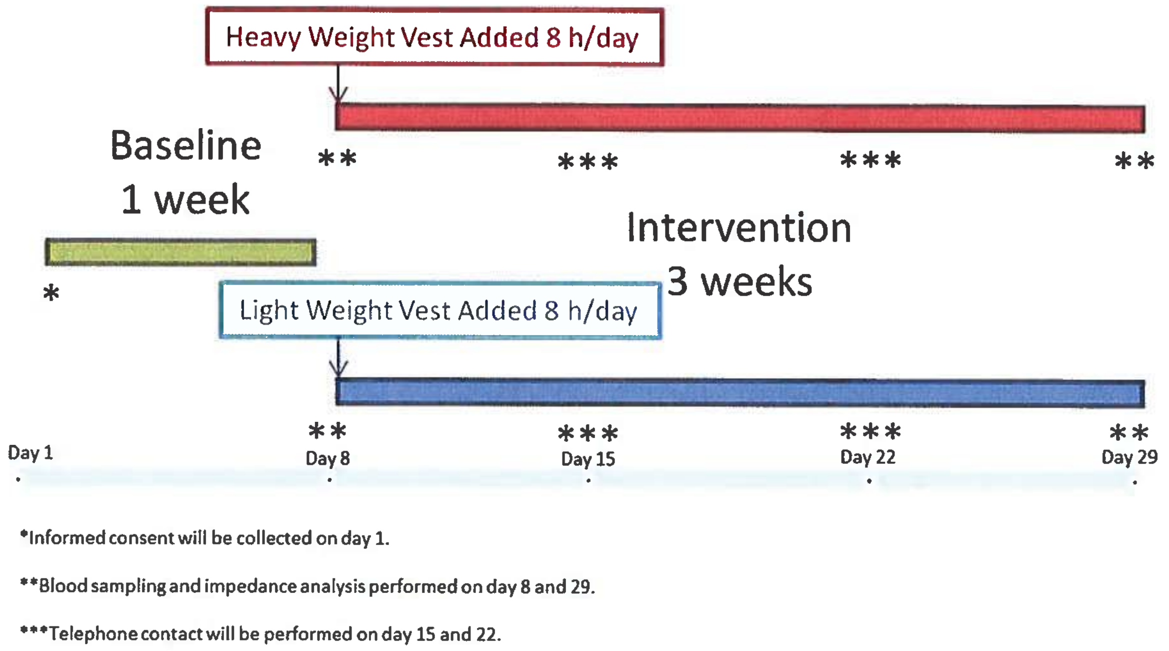

Figure 2. This figure shows the principles of the study. The green line illustrates the first week of the study. Study subjects will have one week to properly learn to count calories and use the pedometers. The red line illustrates the heavy vest group. This group will wear a weight vest with 11 % of body weight for 8 hours each day during three weeks. The blue line illustrates the control group. This group will wear a light weight vest weighing 1 % of body weight for 8 hours each day during three weeks. Calorie intake and physical activity will be measured every study day. *Informing the subject and collecting written consent will be performed on study day 1. **Blood sampling, impedance analysis and contact with medical personnel will be performed on study day 8 and 29. ***Telephone contact with subjects will be performed on study day 15 and 22. This is to prevent mistakes and minimize the loss to follow-up.

The principles of the study are shown in **Figure 2**. The aim of the study is to investigate the effect of adding synthetic weights to obese individuals. The weights will be added as a weight vest with 11 % of the individual's body weight and will be worn for three weeks. Half of the study subjects will be given a weight vest with 11 % of body weight and half of the study subjects will be given a light weight vest. The light weight vest consists of a weight vest with most of the weights removed. The weight of the light vest will be 1 % of body weight giving a difference of 10 % between the groups.

Body weight, fat mass, bone mass, water mass and fat free mass will be measured before and after the intervention by medical personnel to determine the effect of added synthetic weights on obese subject. This will be measured using bioelectrical impedance analysis (BIA) (MC-180MA, Tanita). The scales will be calibrated every week during the study to make certain reliable measurements.

Pedometers will be used to measure the subject's activity each study day. We will also encourage the study participants to report food intake, choice of food and level of hunger each week. This will be done using the validated poll MiniMealQ (9). A pedometer will also be attached to the vests to check the daily use of the vests. The study persons will be informed about this arrangement.

Fasting blood sampling will be performed before and after the intervention to further determine the effects of adding synthetic weights to obese individuals but also to elucidate the mechanism behind the "gravitostat". Blood glucose levels will be measured immediately. Blood samples, except blood glucose, will be analyzed either continuous or after all study subjects have been enrolled depending on human resources.

Study subjects will be recruited by advertising in the local newspaper. Study subjects will be informed about hypothesis that people carrying a heavy or light weight may differ in weight loss. At this point it is not known if the heavy weight vest or the light vest is causing more weight loss, as no one has performed similar studies on humans.

Study subjects will have one week to familiarize themselves with the equipment used in the study before the intervention starts. This is to minimize human errors during the intervention and ensure a high level of compliance. The data collected during this week will be used as baseline.

Day 1 – screening and enrollment

The first 100 persons to contact the researchers and meet all the inclusion criteria and not any of the exclusion criteria will be enrolled in the study. The study subjects will be entitled to a screening visit where study subjects will receive information about the study plan both orally and written during this meeting.

Consumption of more than one liter of wine (11 %) or alcohol equivalent during a study week will not be accepted. Drastic change in lifestyle before or during the study will not be accepted. Any study subject failing to comply with this restriction will instantly be removed from the study.

Education and detailed planning of the study subjects will be performed after written consent is obtained from the study subject. Body weight, fat mass, bone mass, water mass and fat free mass will be measured using BIA. It is unlikely that bone mass and water mass will be affected during the short time of this study but these values are easily obtained during the measurements. We will also measure blood pressure, height in order to calculate BMI and collect each subject's age and gender.

This meeting will end with a blood sample to screen for common diseases that may distort the results and increase the risk of adverse effects. We will measure the following substances in blood: hemoglobin, white blood cells, thrombocytes, sodium, potassium, creatinine, ASAT, ALAT, HbA1c, C-reactive protein (CRP), T4, TSH. Also, we will use urine dipsticks to further screen for diseases. We will measure the following substances in urine: nitrite, white blood cells, red blood cells, glucose, creatinine and albumin. Only subjects showing normal or no clinically significant aberrations of blood and urine analyses will be included in the study.

Study subject will be able to contact the researchers by e-mail or mobile phone any time during the study if questions arise.

Day 8 – intervention starts

Study subjects will be entitled to another appointment with the researchers and the nurse from Gothia Forum one week after the enrollment. Body weight, fat mass, bone mass, fat free mass and water mass will be measured during this visit using BIA.

Fasting blood sampling by an experienced nurse from Gothia Forum will be performed when the researcher believe that the study subject is fully educated and ready to be enrolled in the study. A maximum of 25 ml will be collected at each blood sampling. Plasma levels of glucose will be measured instantly in capillary blood, and thus not be dependent on successful venous blood sampling. Venous blood samples for other measurement than glucose will be centrifuged by the researchers or personnel from Gothia Forum to obtain serum or EDTA plasma samples. Serum and plasma samples may be stored at a temperature of -80°C until analysis. Study subjects can request to have their blood samples destroyed at any time.

The intervention will start directly after blood sampling. Subjects will earn a randomized two- or three-character code to be used throughout the study; this will be noted in a digital only randomization list. Study subjects will obtain either a weight vest with 11 % of body weight or a light vest weighing 1 % of body weight. The vest is to be worn 8 hours each day for the following 3 weeks. The vest is only to be used during awake hours.

Day 15

Study subjects will be entitled to another appointment with the researchers two weeks after the enrollment, this appointment may be performed by telephone if the subject so agree. Any problems with compliance will be discussed. Study subjects who deviate more than 20 % from the study protocol will be removed from the study. Study subjects will continue to wear the vests for 8 hours each day.

Day 22

Study subjects will be entitled to another appointment with the researchers three weeks after the enrollment. This appointment may be performed by telephone if the subject so agree. Any problems with compliance will be discussed. Study subjects who deviate more than 20 % from the study protocol will be removed from the study. Study subjects will continue to wear the vests for 8 hours each day.

Day 29 – intervention ends

Study subjects will be entitled to another appointment in the morning before breakfast with the researchers and the nurse from Gothia Forum four weeks after the enrollment. Body weight, fat mass, bone mass, fat free mass and water mass will be measured during this meeting using BIA. Fasting glucose will be measured in capillary blood. Venous blood sampling will be done. Study subjects will have worn the vests for 8 hours the day before, but they will not wear it during the visit the morning of day 29.

The appointment at day 29 may also be performed on "day 30" depending on the study subject's schedule. Nevertheless, last day for the intervention is on study day 29.

Table 2. This table summarizes all the procedures. Blood samples, except blood glucose, will be analyzed either continuous or after all study subjects have been enrolled depending on human resources.

Table 2 **Study plan detailing the procedures each day**

**Time point (day)**	1	1-8	8	8-15	15	15-22	22	22-29	29
**Window**	+/- 2h	+/- 2h	+/- 2 days	+/- 2h	+/- 2h	+/- 2h	+/- 2h	+/- 2h	+/- 2 days
Height for BMI	X								

Time point (day)	1	1-8	8	8-15	15	15-22	22	22-29	29
**Window**	+/- 2h	+/- 2h	+/- 2 days	+/- 2h	+/- 2h	+/- 2h	+/- 2h	+/- 2h	+/- 2 days
Body weight	X		X						X
Impedance	X		X						X
Fasting Blood Sample			X						X
Blood Glucose			X						X
Screening blood sample	X								
Intervention starts			X						
Intervention ends									X
Intervention (Heavy Weight Vest or Light Vest 8 h/day)				X		X		X	
Reporting Food Intake		X		X		X		X	
Measuring physical activity		X		X		X		X	

3.2. Rationale for study design

We will use a parallel study design in which we compare the placebo group with the intervention group. In order to avoid the risk of carry-over effects, we have decided to not use a crossover design. This could have decreased the variation of the measurement values and increased the power of the study. However, we believe there could be a risk of a carry-over effect with a cross over design. As this is a completely new effect in clinical studies, we have no information about the wash-out time needed to avoid this problem.

4. STUDY POPULATION

4.1. Inclusion criteria

For inclusion in the study, subjects must fulfil the following criteria:

1. Obesity as defined by a BMI >30 and ≤35. Fat mass should be above 25 % (10).
2. 18-70 years of age. We will primarily recruit men. Women will only be recruited if we are unable to find 80 eligible male participants.

3. Consent out of free will.

4. Willingness to comply with the study protocol and restrictions of not consuming excessive amounts of alcohol (maximum 1 litre, 11 % or alcohol equivalent for a full week) or using any drugs. Smoking and snuff use is allowed.

5. Normal or clinically non-significant abberations of screening blood- and urine samples. In blood: hemoglobin, white blood cells, thrombocytes, sodium, potassium, creatinine, ASAT, ALAT, HbA1c, CRP, T4, TSH. In urine: nitrite, white blood cells, red blood cells, glucose, creatinine and albumin.

6. Signed informed consent.

4.2. Exclusion criteria

Subjects must not enter the study if any of the following are present:

1. Chronic disease that hardens the participation in the study as judged by the investigator.

2. Chronic pain such as pain that is constant and impairs quality of life; for example: severe back, hip and knee pain.

3. Regular consumption of medicine or natural supplements that affect weight, inhibit physical activity or increase the risk of adverse effects as judged by the investigator. The following drugs will not be accepted: β-blockers, GLP-1-agonists, SGLT2-inhibitors, sulfonylureas, insulin, orlistat, mysimba and bisphosphonates.

4. Gastric by-pass surgery or equivalent.

5. Reduced mobility.

6. Pregnancy: Females of childbearing potential must confirm to use reliable contraception and not suspect to be pregnant. Subjects may be asked to perform a pregnancy test.

7. Change in body weight of 5 kg or greater during the past 3 months or recently started a strict diet. Also, a greater change in body weight than 1.5 kg difference between day 1 and day 8 will not be accepted.

8. Drastic change in lifestyle during the last 3 months; for example a significant change in physical activity or nicotine, alcohol or drug use.

9. Apparent risk of not being able to comply with the study protocol for any reason as judged by the investigator.

Reasons for exclusion of participants will be reported in the publication.

4.3. Restrictions

The study subjects will not be allowed to remove the vests for 8 hours upon putting it on. Subjects who deviate more than 20 % from this (i.e. use the vest for less than 6.4 hours during the daily 8 hour vest wearing period) will be excluded from the study.

The study subjects are only allowed to consume a limited amount of alcohol and other drugs during the study. This is in line with the inclusion- and exclusion criteria.

4.4. Subject enrolment and randomization

We will recruit healthy individuals by advertising in local newspapers. We will only enroll healthy people who do not know the hypothesis of the study. Each study subject will be given a randomized code to use for the study. It will then be decided by dice throw if a study subject is to be included in the group wearing heavy weight vests or light weight vests. Subjects will be placed in the group with heavy weight vests if the dice shows 1,2 or 3 and in the group with light weight vests if the dice shows 4, 5 or 6. Due to the risk of drop-outs we will aim to recruit 50 participants in each group for a total of 100 study subjects. The exact number of screened patients depend on the frequency of screening failure; it is likely that we need to screen more than the double number of subjects needed. 40 participants are needed in each group to receive a power of 80 % to discover a difference in body weight change of ≥ 1.6 %.

The last participants to enroll in the study may be placed in the group with the least number of study subjects by the researchers if a total of 50 study subjects already have been enrolled in one of the groups. Only the study subject and the investigator will be able to connect each study subject with their randomized code. Identification of individual study subjects will only be performed under special circumstances, for example, if a study subject requests removal of his or her study data.

Study subject eligibility will be established before enrolment and randomization. Study subjects will be randomized strictly sequentially, as study subjects are eligible for randomization. If a study subject discontinues from the study, the study subject number will not be reused, and the study subject will not be allowed to re-enter the study.

4.5. Discontinuation and withdrawal of subjects

Subjects are free to discontinue their participation in the study at any time without prejudice to further treatment. The subjects may be withdrawn from the study at the discretion of the investigator due to safety concerns or if judged non-compliant with study procedures. In either case, serious adverse events will be followed up. Other reasons for discontinuing a subject are incorrect enrolment and subjects lost to follow-up.

4.5.1. Premature termination of the study

The research group may decide to stop the trial or part of the trial at any time. If a trial is prematurely terminated or suspended, the investigator should promptly inform the study subjects. Furthermore, the investigator should promptly inform the Ethics Committee and provide a detailed written explanation.

5. STUDY TREATMENTS

No drugs or medicinal treatment are used in this study. However, study subjects are obliged to wear a weight vest with 11 % of body weight. As discussed previously we judge that this is a study with few risks and only mild potential adverse effects.

6. STUDY MEASUREMENTS AND VARIABLES

6.1. Variables

The primary variable to be measured will be the change of body weight in % of the baseline body weight.

The following parameters are measured during the experiment:

1. Bioelectrical impedance analysis measurement at study day 1, 8, and 29.

Body weight will be measured (kg) which will be used to calculate the change in bodyweight (kg) between the measurements. We will also measure the study subject's height in order to calculate changes in BMI.

Bioelectrical impedance analysis measurement will also be used to calculate change in fat mass (kg), bone mass (kg), fat free mass (kg) and water mass (kg).

2. Blood samples at study day 1, 8, and 29.

Measurements by the Sahlgrenska Academy, University of Gothenburg: insulin, HDL, LDL, TAG, FGF21, osteocalcin, FGF23, FGF15, sclerostin, lipocalin2, leptin, ghrelin, GLP-1, GLP-2, CCK, (3-36)PYY, glucagon, testosterone, estrogens, renin and methoxynorepinephrine (Day 8 and day 29).

Measurements by Sahlgrenska University hospital (central lab and Clinical Trial Center): blood glucose (Clinical Trial Center) and circulating Na, K, Cl, Ca and creatinine (C-Lab). Sahlgrenska University hospital will also measure the day 1 screening blood- and urine samples. This includes the following samples in blood: hemoglobin, white blood cells, thrombocytes, Na, K, creatinine, ASAT, ALAT, HbA1c, CRP, T4, TSH. Urine samples will be analyzed using urine dipstick for the following substances: nitrite, white blood cells, red blood cells, glucose, creatinine and albumin.

3. Self-reported food intake. Choice of food, energy (kcal) and hunger will be assessed.

4. Physical activity will be monitored by pedometers. Energy expenditure by physical activity (kcal), and total mobility (walking distance, steps taken) will be assessed.

6.2. Biological sampling procedures

6.2.1. Handling, storage and destruction of biological samples

To determine the mechanism of action and elucidate the beneficial effects of body weight loading we will analyze blood samples. Some tubes may be used for analyses by Sahlgrenska University hospital central lab and the rest of the tubes will be used for analyses by the research group on site or at the Institute of Neurology and Physiology. All samples will be impossible to connect to a study subject without knowing their randomization code. Measurements will be performed as quickly as possible and samples will then be destroyed. At any time, a study subject can request destruction of his or her blood samples.

Each venous blood drawing consists of a maximum of 25 ml. Every sample will then be divided in four tubes – each consisting of 5-6 ml for measurements. The tubes used for blood drawings will contain ethylenediaminetetraacetic acid (EDTA) or a SST-gel for the purpose of manipulating coagulation.

Fasting blood glucose levels will be measured directly in capillary blood using a glucometer (Hemocue®).

The samples may be stored at the Sahlgrenska Academy before the actual measurements because of practicalities. All samples will be stored anonymized with only the randomization code on it at a temperature of -80°C. This has been reported to the Swedish "health and Social Care Inspectorate" according to regulation 2002:297.

If we experience technical difficulties measuring any of the above parameters some samples may be sent to another EU/EES country for measurements. The samples will of course be anonymized during the full process. The analysis may take place at a cooperative group, the industry or drug manufacturer. The samples will be returned or destroyed upon completed analysis.

6.2.2. Total volume of blood drawn per patient

Each blood sampling will consist of approximately 25 ml blood and be divided into four tubes. It means that in total a maximum of 75 ml blood will be taken during the study. In comparison, a blood donation corresponds to ca 450 ml.

7. SAFETY

In general this is a study with few risks. The weight vests used in this experiment are daily used by thousands of people for exercise. During previous experiments on more than 70 medical students only one of the students were affected with an adverse event. This student experienced mild dizziness which subsided within a few minutes after removing the weight vest and sitting down. However, it should be noted that the risks of wearing a weight vest for several weeks in a row are largely unknown.

To minimize the risks of our future study we have decided to include only healthy research subjects not consuming any or few medications. We will also have medical trained personnel available during the study and perform it in close proximity to the Sahlgrenska University Hospital.

Blood sampling can be experienced as somewhat uncomfortable by a few individuals. However it is generally free from complications. Occasionally, there may be small local bruising and local inflammation. We may fail to get venous blood from 5-15% of these obese subjects. They will still be part of the study, and their levels of the primary outcome body weight will be included in the study.

If an adverse event occurs the study subject can abort the study whenever he or she wants to. The medical personnel involved in the study will help the subject to the correct medical care giver practice if needs arises. Adverse events will be reported in the publication.

8. STATISTICS

8.1. Sample size calculation

Based on our extensive data demonstrating that increased loading decreases body weight in mice and rats (3) we will perform a randomized interventional study to determine if increased loading decreases body weight also in humans. Subjects will be randomized to treatment with either (I) increased loading by using a Casall HIT weight vest that weighs 11 % of the baseline body weight (=High load-treated) or (II) essentially unchanged loading by using a vest with much lower weight (1% of baseline body weight, = Low load treated). The experimental subjects will carry the vest for 8 awake hours per day for 3 weeks.

The primary endpoint of this randomized study will be change in body weight expressed as percent of original weight between day 8 and 29, i.e. the days of the intervention. These values will be calculated for all individuals participating in the study. These values will then be compared between the groups of individuals exposed of 11% and 1 % extra loading, using student's t-test (assuming normality). We aim to include at least 40 subjects in each treatment group, resulting in 80 % power to discover a difference in body weight change of ≥ 1.6 %. A total of 100 study subjects will be recruited to achieve a minimum of 40 subjects in each group.

Inclusion criteria consists of but is not limited to BMI 30-35 kg/m2, relatively stable body weight during the last three months (less than five kg change and less than 1.5 kg measured change during the first week of the study), age 18-70 years and that the individuals are essentially healthy. The full list of inclusion and exclusion criteria can be found at section 4.1 and 4.2. The plan is to primarily include men because it has been shown that their variability in body weight measured at 3-5 week intervals is less than for women (SD 1.0 kg for men, 1.3 kg for women).

Secondary endpoints will include change in fat mass, bone mass, water mass and fat free mass measured with bioimpedance, serum glucose levels, serum leptin levels and possibly also serum levels of the putative body weight regulating endocrine factor released from osteocytes. Potential body weight regulating factors are listed at section 6.1. Furthermore, we will assess change in food intake (kcal) and physical activity (kcal, walking distance, steps taken).

8.2. Statistical analysis

Student's t-test in SPSS Statistics will be used to compare the two groups if normally distributed before or after logarithmical transformation.

A non-parametric analysis, Mann Whitney U test, will be performed if data cannot be assumed normally distributed even after logarithmical transformation.

An analysis of covariance (ANCOVA) using age, BMI and sex as co-variates will also be performed.

One calculation will be used for each of the endpoints although we only have one primary endpoint.

Subjects who have used the weight vest 3 SD less than mean will be excluded from the analysis.

9. DATA MANAGEMENT

9.1. Recording of data

The investigator will ensure that all data collected in the study are recorded in a timely manner according to any instructions provided.

An electronic Case Report Form will be used for data collection. The investigator will ensure that the data are recorded in the Case Report Form as specified in the study protocol and in accordance with the instructions provided. The investigator ensures the accuracy, completeness and timeliness of the data recorded. The investigator will sign the completed Case Report Form. A copy of the completed Case Report Form will be archived at the study site, in this case The Sahlgrenska Academy.

9.1.1. Source data

The following documents comprise the source data and will be recorded in the Case Report Form at the study site:

- An individual procedure protocol which is an excel file consisting of each study subjects reported calorie intake, physical activity and the results of the bioelectrical impedance analysis measurements. Gender, length, inclusion criteria and other specific information for that particular study subjects will also be included in this file.
- The measurement values obtained from Sahlgrenska University hospital central lab.
- The measurement values obtained from the analyses at the Sahlgrenska Academy.
- A copy of the informed consent from each study subject.

9.2. Data storage and management

All data should be recorded, handled and stored in a way that allows its accurate reporting, interpretation and verification. All raw data, a copy of the completed Case Report Form, original protocol with amendments and the final report will be stored for a minimum period of ten years after termination of the trial, in accordance with Swedish regulation/law (Chapter 10, 3 § in LVFS 2011:19).

At the conclusion of the study, the occurrence of any protocol deviations will be determined. After these actions have been completed and the database has been declared to be complete and accurate, it will be locked and available for data analysis.

10. QUALITY CONTROL AND QUALITY ASSURANCE

10.1. Monitoring

The investigators will have close contact to Gothia Forum during the entire study until the publication; this is to ensure that the investigators are appropriately trained and informed about patient written information and applicable regulatory requirements. The research group may also consult external experts to verify and correct the data analyses if needed. The study will be monitored by Gothia Forum according to a less detailed schedule (not according to good clinical practice) as no drug or medical device is used. The monitor will before, during and after the study control regulatory documents, informed consent, handling of unexpected adverse events and the primary endpoint data.

10.2. Audits and inspections

Authorized representatives of an Ethics Committee may perform audits or inspection at the institution, including source data verification. The purpose of an audit or inspection is to systematically and independently examine all study-related activities and documents, to determine whether these activities were conducted, and data were recorded, analyzed and accurately reported according to the protocol, Good Clinical Practice and any applicable regulatory requirements.

11. ETHICS

The study will be performed in accordance with ethical principles that have their origin in the Declaration of Helsinki and applicable regulatory requirements. However, monitoring of the study will not fulfill all criteria which are consistent with good clinical practice as no drug or medical device is used.

11.1. Ethics committee

The final study protocol, including the final version of the Informed Consent Form and other information given to subjects must be approved or given a favorable opinion in writing by an Ethics Committee as appropriate. The Principal Investigator is responsible for informing the Ethics Committee of any amendment to the protocol, in accordance with local requirements.

11.2. Informed consent

The Principal Investigator will ensure that the subject is given full and adequate oral and written information about the nature, purpose and possible risks and benefits of the study. Subjects must also be notified that they are free to discontinue from the study at any time. The subject should be given the opportunity to ask questions and allowed time to consider the information provided. The subjects will also be able to contact the researchers using phone or e-mail at any time during the study period.

The subject's signed and dated informed consent must be obtained before conducting any procedure specifically for the study.

The original, signed Informed Consent Form must be stored in the Investigator's Study File. A copy of the signed Informed Consent Form must be given to the subject.

If a protocol amendment requires a change to the Informed Consent Form, the Ethics Committee must approve modifications that lead to a revised Informed Consent Form before the revised form is used.

11.3. Subject data protection

The Informed Consent Form will incorporate wording that complies with relevant data protection and privacy legislation. Pursuant to this wording, subjects will authorize the collection, use and disclosure of their study data by the investigators.

The Informed Consent Form will explain that study data will be stored in a computer database, maintaining confidentiality in accordance with national data legislation. All data processed by the investigators will be identified by a code.

11.4. Insurances and compensation

The study subjects are insured by patient injury insurance. A compensation of 500 SEK will be payed to each research subject.

12. PROTOCOL DEVIATIONS AND AMENDMENTS

Modifications to the signed protocol are only possible through approved protocol amendments and with the agreement of all responsible persons. Details of non-substantial amendments are to be clearly noted in the amended protocol. In case of a substantial protocol amendment, the concerned Ethics Committee must be informed and should be asked for its approval prior implementation of amended protocol, as to whether a full re-evaluation of the ethical aspects of the study is necessary by the committee. This should be fully documented.

13. REPORT AND PUBLICATIONS

After completion of the study, the results will be analyzed and a study report will be prepared. In addition, upon study completion and finalization of the study report the results of this trial will be either submitted for publication or posted in a publicly accessible database of clinical trial results.

14. STUDY TIMETABLE

The study will take place between Q3 2018 and Q1 2019. All analyzes shall be finished before 2022-12-31. Publications of the results are planned for late 2019, but may not happen until 2022.

15. LIST OF REFERENCES

1. WHO. Obesity and overweight http://www.who.int/mediacentre/factsheets/fs311/en/: WHO; 2016 [updated June 2016]. Available from: http://www.who.int/mediacentre/factsheets/fs311/en/.
2. Katzmarzyk PT, Church TS, Craig CL, Bouchard C. Sitting time and mortality from all causes, cardiovascular disease, and cancer. Medicine & Science in Sports & Exercise. 2009;41(5):998-1005.
3. Jansson J-O, Palsdottir V, Hägg DA, Schéle E, Dickson SL, Anesten F, et al. Body weight homeostat that regulates fat mass independently of leptin in rats and mice. Proceedings of the National Academy of Sciences. 2018;115(2):427-32.
4. Cannon W. The Wisdom of the Body. 312 pp. New York. 1932.
5. Zhang Y, Proenca R, Maffei M, Barone M, Leopold L, Friedman JM. Positional cloning of the mouse obese gene and its human homologue. Nature. 1994;372(6505):425.
6. Friedman J. 20 years of leptin: leptin at 20: an overview. Journal of endocrinology. 2014;223(1):T1-T8.
7. Frederich RC, Hamann A, Anderson S, Löllmann B, Lowell BB, Flier JS. Leptin levels reflect body lipid content in mice: evidence for diet-induced resistance to leptin action. Nature medicine. 1995;1(12):1311.
8. Montague CT, Farooqi IS, Whitehead JP, Soos MA, Rau H, Wareham NJ, et al. Congenital leptin deficiency is associated with severe early-onset obesity in humans. Nature. 1997;387(6636):903.
9. Christensen SE, Möller E, Bonn SE, Ploner A, Wright A, Sjölander A, et al. Two new meal-and web-based interactive food frequency questionnaires: validation of energy and macronutrient intake. Journal of medical Internet research. 2013;15(6).
10. Larsson I, Forslund HB, Lindroos A, Lissner L, Näslund I, Peltonen M, et al. Body composition in the SOS (Swedish Obese Subjects) reference study. International journal of obesity. 2004;28(10):1317.

Statistical Analysis Plan

Study: Effect of different weight vests on body weight in obese individuals

Code: EVO

Version: 1.0

Primary endpoint

The primary endpoint of this randomized study will be change in body weight expressed as percent of original weight between day 8 and 28, i.e. the days of the intervention. The values will be calculated for all individuals participating in the study. The values will then be compared between the groups of individuals exposed of 11% and 1 % extra loading, using student's t-test (assuming normality).

- Student's T-test will be used to compare the two groups if normally distributed before or after logarithmical transformation.
- A non-parametric analysis, Mann Whitney U test, will be performed if data cannot be assumed normally distributed even after transformation.
- An analysis of covariance (ANCOVA) using age, BMI and sex as co-variates will also be performed.

Secondary endpoints

The secondary endpoints of this study are found under section 6.1 in the study protocol. All the secondary endpoints are regarded as exploratory endpoints. These will be analyzed using the same methods as described for the primary endpoint. We will also compare values at the end of the study and absolute changes between the groups of individuals exposed of 11% and 1 % extra loading.

Exclusions

Subjects who have used the weight vest 3 SD less than mean will be excluded from the analysis, this will be assessed using pedometres. Data from subjects who have not finished the full intervention will also be removed from the final calculation. However, it is possible that we fail to draw blood from some of the subjects, these subjects will not be excluded from the study.

This document was written before any data were collected

J-OJ

....

John-Olov Jansson, Professor

Gothenburg 2018-09-25

Statistical Analysis Plan

Study: Effect of different weight vests on body weight in obese individuals

Code: EVO

Version: 1.1 (2018-12-19)

Primary endpoint

- The primary endpoint of this randomized study will be body weight at the end of the intervention (day 28) expressed as percent of original weight at day 8, i.e. the day of the start of the intervention. The values will be calculated for all individuals participating in the study.
- The values will then be compared between the groups of individuals exposed of 11% and 1 % extra loading, using analysis of covariance (ANCOVA) assuming normality.
- The ANCOVA may be performed using age, BMI at the start of the study, sex, time per day wearing the west, and percent of the vest bearing time standing up as co-variates. The co-variates will be included if they are found to be confounders.
- Non-parametric analysis will be performed if data cannot be assumed normally distributed even after transformation.

Secondary endpoints

The secondary endpoints of this study are found under section 6.1 in the study protocol. All the secondary endpoints are regarded as exploratory endpoints. These will be analyzed using the same methods as described for the primary endpoint. We will also compare values at the end of the study and absolute changes between the groups of individuals exposed of 11% and 1 % extra loading.

Exclusions

Subjects who have used the weight vest 3 SD less than mean or less than 80% of requested time will be excluded from the analysis., this will be assessed using self-reporting . Data from subjects who have not finished the full intervention will also be removed from the final calculation. However, it is possible that we fail to draw blood from some of the subjects, these subjects will not be excluded from the study.

This document was written before the data base was locked

....

John-Olov Jansson, Professor

Gothenburg 2018-12-20
